# Molecular Orientation Controls Triplet Exciton Dynamics in Organic Molecules Coupled to Lanthanide-Doped Nanoparticles

**DOI:** 10.1021/jacs.5c13962

**Published:** 2025-10-02

**Authors:** Lars van Turnhout, Alasdair Tew, Simon A. Dowland, Ebin Sebastian, Zhao Jiang, Rakesh Arul, Zhongzheng Yu, Akshay Rao

**Affiliations:** † Cavendish Laboratory, 2152 University of Cambridge, Cambridge CB3 0US, United Kingdom

## Abstract

Hybrid organic–inorganic materials, which combine the unique properties of organic semiconductors (OSCs) and inorganic nanoparticles, show great promise for optoelectronic applications. Understanding structure–function relationships in these nanohybrids is crucial for understanding the mechanisms governing their excited state dynamics, particularly those controlling triplet excitons, which are key to their performance. While previous studies focused primarily on triplet energy transfer (TET) across the interface, we study the full triplet exciton dynamics in OSCs coupled with various lanthanide-doped nanoparticles (LnNPs), with an emphasis on the impact of molecular orientation. We examine three anthracene carboxylic acid (ACA) isomers, differing in the position of the carboxylic acid group (1-, 2-, and 9-position) on the anthracene core. These isomers all exhibit similar triplet dynamics and low triplet yields (∼10%) when uncoordinated, but adopt distinct binding geometries on the LnNP surface, making them ideal for studying how coordination geometry influences triplet exciton dynamics. Using time-resolved optical spectroscopy, we observe significant variations in triplet generation rates, yields, lifetimes, and TET rates between the ACA isomers upon coordination onto the LnNPs. Triplet generation rates and yields are consistently highest in 1-ACA (up to 86%) and lowest in 2-ACA. TET rates are fastest for 9-ACA (up to 1.1 × 10^8^ s^–1^) and slowest for 2-ACA. Notably, in the absence of TET, triplet exciton lifetimes exceed 0.1 ms for all LnNP@ACA nanohybrids. These results quantitatively describe how positional isomerism governs the triplet exciton dynamics in LnNP@OSC nanohybrids and highlight the pivotal role of molecular orientation in mediating interfacial photophysics.

## Graphical abstract

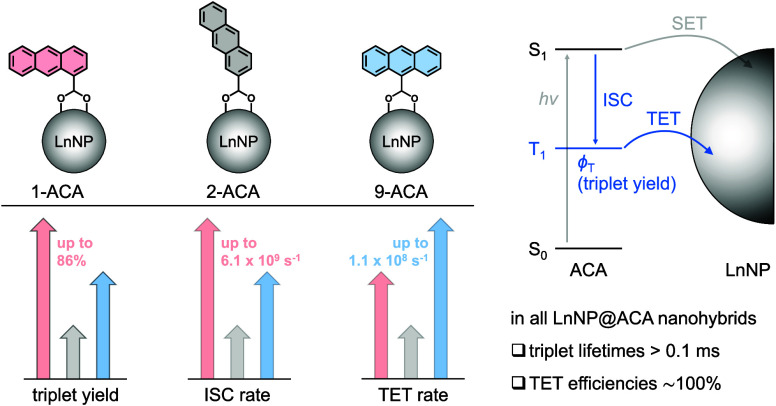

## Introduction

Triplet excitons are of great importance for applications in optoelectronics,^1–4^ bioimaging,^5,6^ photocatalysis,^7,8^ and photon upconversion.^9,10^ Compared to spin-0 singlet excitons, spin-1 triplet excitons exhibit long lifetimes and large diffusion lengths, enabling them to act as long-range energy carriers and reservoirs.^11^ Despite the potential of triplet excitons, their generation and luminescent harvesting are obstructed by their spin-physics as direct optical transitions between singlet and triplet states are quantum mechanically forbidden, i.e., triplets are considered ‘dark states’.^12^

Various approaches have been developed to overcome the limitation of triplets being dark states. The most well-known approach is to generate and optically harvest triplet excitons via heavy-metal induced spin–orbit coupling (SOC), where the inclusion of heavy metals partially lifts spin-selection rules.^13,14^ Another approach is to tune the singlet–triplet energy gap to allow for thermally activated delayed fluorescence (TADF).^15,16^

Over the past decades, triplet exciton control at organic–inorganic interfaces, in which organic semiconductors (OSCs) are coordinated onto inorganic nanoparticles, has been studied increasingly. Having a detailed comprehension of the hybrid interface is crucial for designing structures that efficiently harvest and convert solar light. This has been illustrated by for example perovskite and dye-sensitized solar cells,^17–21^ as well as by organic–inorganic upconversion systems.^4,9,22,23^

There has been significant interest in studying the triplet exciton dynamics in OSC–quantum dot (QD) systems, with anthracene carboxylic acid (ACA) frequently employed as a model OSC.^3,22,24–26^ Some of these studies explored the role of molecular orientation, demonstrating the importance of the position of the carboxylic acid group on ACA in mediating interfacial triplet energy transfer (TET). For example, photon upconversion efficiencies of CdSe QDs varied by more than 10-fold depending on the specific ACA isomer. Another study examining the interaction between two ACA isomers and CdSe QDs found the TET mechanism to be either through-bond (2-ACA) or through-space (9-ACA), with a through-space mechanism favored for smaller CdSe nanocrystals with larger wave function leakage.^27^ While these results highlight the importance of binding geometry, prior work has focused exclusively on TET and has not addressed how coordination geometry affects the broader triplet exciton dynamics.

In recent years, OSCs coupled with inorganic lanthanide-doped nanoparticles (LnNPs) have emerged as a new organic–inorganic platform to control triplet excitons.^4,28–30^ In these systems, OSCs with high molar absorption coefficients act as sensitizers for the LnNPs, which have inherently low absorption cross sections.^31^ It has been shown that the interaction of OSCs with LnNPs can enhance triplet exciton generation.^4,29^ Furthermore, efficient TET to the LnNPs through a Dexter-type process has been demonstrated.^32–34^ Yet, to the best of our knowledge, no studies have investigated how the coordination geometry of OSCs on the LnNP surface affects the triplet exciton dynamics in these systems.

In this work, we examine how the molecular orientation of OSCs on the surface of LnNPs affects and can be used to control the triplet exciton dynamics in LnNP@OSC nanohybrids. For this study, we use three positional ACA isomers: 1-ACA, 2-ACA, and 9-ACA, as shown in Figure 1, where the carboxylic acid group that coordinates onto the LnNPs is located at different positions on the anthracene core. These isomers exhibit distinct orientations when attached to the LnNPs, allowing for a controlled change in the orientation of the molecular wave function with respect to the LnNP surface, which we hypothesize will result in variations in the triplet exciton dynamics. The uncoordinated ACA isomers show similarly low intrinsic triplet yields in solution, ∼10%, thus offering an ideal system to probe the effect of molecular orientation on the triplet dynamics upon coordination onto LnNPs.

**Figure 1:**
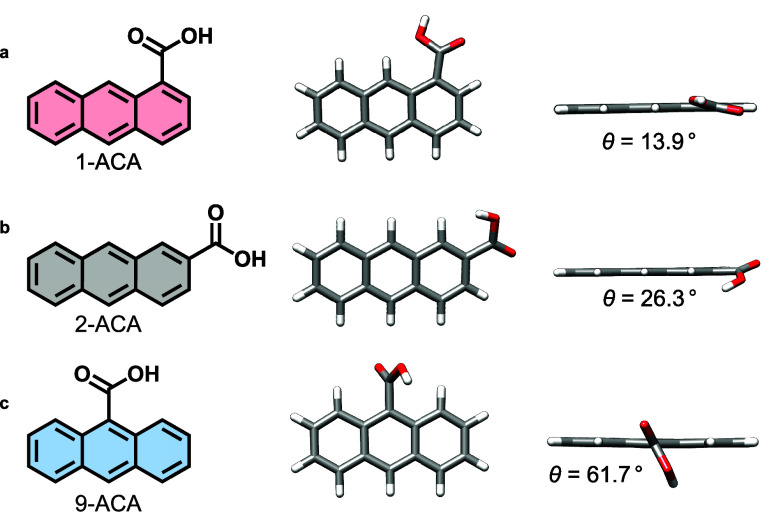
(a–c) Molecular structures and optimized geometries of (a) 1-ACA, (b) 2-ACA, and (c) 9-ACA, with the corresponding torsion angles (*θ*) along the carboxylic group in the ground electronic state.

Using a range of optical spectroscopies, we provide a comprehensive overview of the triplet exciton dynamics, including triplet generation rates, triplet yields, triplet lifetimes, TET rates, and TET efficiencies in various LnNP@ACA nanohybrids. Upon coordination, we consistently find triplet exciton yields and triplet generation rates through intersystem crossing (ISC) to be highest for 1-ACA and slowest for 2-ACA. We find that triplet exciton yields increase from ∼10% for uncoordinated ACA isomers, to up to 43% for 2-ACA once coordinated, and up to 86% for 1-ACA after coordination onto various LnNPs. TET rates, on the contrary, are highest for 9-ACA and lowest for 2-ACA, with TET rates being up to 22 times faster for 9-ACA than 2-ACA under identical conditions. Interestingly, in systems in which no TET occurs, despite accelerations in triplet generation resulting in triplet yields of up to 86%, we still find triplet exciton lifetimes well beyond 0.1 ms, opening avenues for applications in photocatalysis or phototherapeutics that rely on long-lived excited states. Overall, this work quantitatively maps structure–function relationships in LnNP@ACA nanohybrids, offering new insights into how molecular orientation governs triplet exciton dynamics at hybrid organic–inorganic interfaces.

## Results

### Fabrication of LnNP@ACA Heterostructures

LnNPs (Ln = Y, Eu, Gd, Yb) of the type NaLnF_4_ were synthesized according to a known procedure by Wang et al.^35^ A detailed synthetic description can be found in the Supporting Information. The as-synthesized particles were prepared with oleic acid (OA) as surface ligands. TEM images revealed the LnNPs to have mean diameters of 5.2 ± 0.5 nm (YNPs), 5.1 ± 0.5 nm (EuNPs), 5.0 ± 0.4 nm (GdNPs), and 4.8 ± 0.4 nm (YbNPs) (Figure S1). Care was taken to ensure all LnNPs had similar sizes to allow for fair comparisons between them.

To prepare the LnNP@ACA nanohybrids, a ligand exchange was performed in a THF/toluene mixture with the ACA isomers, where the ACA ligands partially replaced the native OA ligands, as described in the Supporting Information. Figure 2a shows the structure of the LnNP@ACA nanohybrids and the expected coordination geometry and orientation of each of the ACA isomers. We calculated the torsion (dihedral) angles θ for the three structures and found that for the ground state, θ increases from 13.9° (1-ACA), to 26.3° (2-ACA), to 61.7° (9-ACA), due to increased steric crowding, as shown in Figure 1. Additional calculations for the deprotonated carboxylate states of the isomers (ACA^–^) show modest reductions in the torsional distortion, but the torsion angles show similar trends between the three isomers (Figure S7). In the excited state, torsion angles for both the protonated and deprotonated states approach 0° (planarity), with larger planarization observed for the deprotonated forms (Figure S8). We assume these trends to be preserved upon coordination.

**Figure 2:**
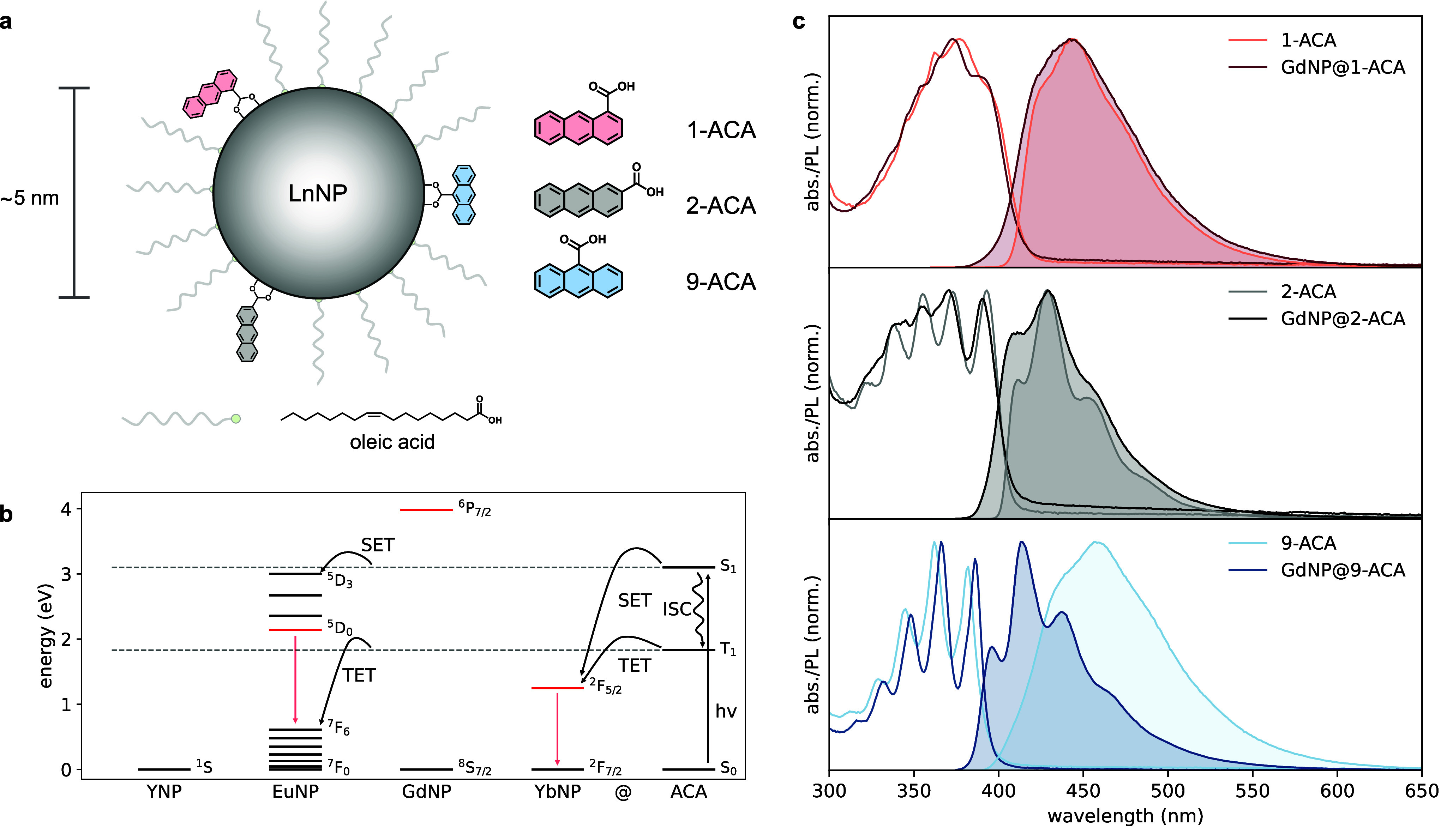
(a) Molecular structures of the ACA isomers and a schematic illustration of their coordination geometry onto LnNPs following partial replacement of the native OA ligands. (b) Relevant energy levels of the ACA isomers and Ln­(III) ions in LnNPs. Following excitation of ACA to generate the S_1_ state, this state can undergo ISC to produce the T_1_ state. For EuNPs and YbNPs, SET and TET can occur. (c) Absorption (line) and PL (shaded) spectra of the ACA isomers (lighter line) and GdNP@ACA (darker line) nanohybrids.

We observed that the different ACA isomers replaced varying amounts of OA during the ligand exchange, with 1-ACA replacing more OA than 2-ACA, and 2-ACA replacing more than 9-ACA. We hypothesize the different amounts of OA being replaced by the various ACA isomers result from the coplanarity of the carboxylic group, which facilitates easier penetration into the LnNP ligand shell and more efficient OA replacement for 1-ACA and 2-ACA than 9-ACA. Therefore, different amounts of each ACA isomer were used during the ligand exchange to achieve comparable ligand coverages for each isomer, ensuring that any observed differences in triplet exciton dynamics upon coordination were not caused by variations in ACA loading on the LnNP surface.

Using the molecular extinction coefficients of the ACA isomers, we optimized the ACA loading to approximately 0.1 mg ACA per 15 mg LnNP, which corresponds to ∼6–7 ligands per LnNP, resulting in a ligand coverage of 0.09 ACA/nm^2^ for all LnNPs. A relatively low ligand coverage was chosen to minimize ligand–ligand interactions on the LnNP surface, as such interactions have been reported to lead to bimolecular annihilation processes,^36^ resulting in undesired deactivation of the triplet excitons and complicating the analysis. The amounts of ACA used for each ligand exchange, molar extinction coefficients, and ligand coverage calculations are summarized in the Supporting Information.

Figure 2b shows the energy levels of the S_1_ (∼3.1 eV) and T_1_ (∼1.8 eV) states of the ACA isomers, as well as those of the four LnNPs used in this study. The S_1_ energies of the ACA isomers were determined from the intersection of the absorption and emission spectra to be 3.01 eV (1-ACA), 3.07 eV (2-ACA), and 3.09 eV (9-ACA) for the uncoordinated ACA isomers, and 3.05 eV (1-ACA), 3.11 eV (2-ACA), and 3.16 eV (9-ACA) for the coordinated ACA isomers. The T_1_ energies of the ACA isomers were determined through phosphorescence measurements. Phosphorescence spectra were measured for GdNP@ACA nanoconstructs, and the T_1_ energies, as determined from the highest energy maxima in the phosphorescence spectra, were found to occur at ∼1.85 eV for all ACA isomers, consistent with previous reports.^37,38^ These results show that the S_1_ and T_1_ energies of the three ACA isomers are nearly constant, allowing for fair comparisons between them.

YNPs and GdNPs were selected for this study because they lack energy levels capable of accepting energy from the ACA isomers, thereby allowing for monitoring of the triplet exciton dynamics in the absence of energy transfer. In contrast, EuNPs and YbNPs possess energy levels that facilitate both singlet energy transfer (SET) and TET. For EuNPs, exergonic SET occurs to the ^5^D_0_ – ^5^D_3_ energy levels (2.14–3.02 eV), while exergonic TET is limited to the dark ^7^F_J_ (0.61–0 eV) manifold. Additionally, back energy transfer (BET) from the emissive ^5^D_0_ level (2.14 eV) to the ACA T_1_ states is possible. For YbNPs, both SET and TET can exclusively occur to the emissive ^2^F_5/2_ energy level (1.25 eV).

Figure 2c shows the absorption and emission spectra of the uncoordinated ACA isomers and GdNP@ACA nanohybrids. The bathochromic shift in the absorption edge of pristine 1-ACA and 2-ACA, compared to 9-ACA, is attributed to the enhanced resonance between the anthracene core and the carboxylate group, which is more coplanar for the ground state of 1-ACA and 2-ACA, while being more perpendicular for 9-ACA (Figure 1).^39^ Successful attachment of the ACA isomers was confirmed by UV–vis absorption spectroscopy, where the ACA absorption features are present on top of the GdNP scattering background, along with a characteristic shift in the ACA vibronic progressions post coordination. Similar shifts were noted for all LnNPs, as shown in Figure S2.

The photoluminescence (PL) spectra of the uncoordinated ACA isomers and the GdNP@ACA nanohybrids are shown in Figure 2c. Little fine structure is observed in the PL spectrum of 1-ACA, which is characteristic of the carboxylic acid group being coplanar with the anthracene ring.^25^ The PL spectrum of uncoordinated 9-ACA is much broader and red-shifted in comparison to that of 1-ACA and 2-ACA. This is consistent with previous studies reporting structureless, broad PL from 9-ACA in aprotic solvents, which has been ascribed to the formation of intermolecular hydrogen-bonded dimers and higher-order aggregates.^40,41^ Upon coordination, the vibronically structured PL of 9-ACA is recovered, consistent with the suppression of aggregate contributions. This recovery of the vibrational fine structure further confirms successful attachment of the ligands. The reduced Stokes shift indicates that the observed PL originates from monomeric, surface-bound 9-ACA. Similar PL spectra were observed for all LnNP@ACA nanohybrids (Figure S3).

### Triplet Exciton Dynamics in Uncoordinated ACA Isomers

Time-resolved PL measurements were performed on the uncoordinated ACA isomers, yielding observed fluorescence lifetimes of 5.8 ns (1-ACA), 9.8 ns (2-ACA), and 10.2 ns (9-ACA). The similar fluorescence lifetimes observed for 2-ACA and 9-ACA are in good agreement with their measured PLQEs: 30.2% (1-ACA), 44.8% (2-ACA), and 44.4% (9-ACA). Using these measurements, we calculated the radiative and sum of nonradiative rate constants for each ACA isomer, a summary of which is provided in Table S1.

While time-resolved PL measurements provide insights into the excited state kinetics, they do not offer direct information about the triplet exciton dynamics. Therefore, we turned to pump–probe spectroscopy. We performed triplet sensitization experiments using PdOEP as a triplet sensitizer (Figure S9). These measurements revealed the T_*n*_ ← T_1_ photoinduced absorption (PIA) of 1-ACA as a broad spectral feature centered around 450 nm, whereas the T_*n*_ ← T_1_ PIAs of 2-ACA and 9-ACA appeared as sharper features between 430–440 nm and 420–430 nm, respectively. From the sensitization measurements, we calculated the T_*n*_ ← T_1_ PIA extinction coefficients for each ACA isomer, which were found to be 8.5 × 10^3^ M^–1^ cm^–1^ (1-ACA), 13.4 × 10^3^ M^–1^ cm^–1^ (2-ACA), and 10.3 × 10^3^ M^–1^ cm^–1^ (9-ACA) at their respective peak absorption wavelengths, see the Supporting Information (Table S4) for a detailed description. We used these values to calculate the triplet yields further on.

We next measured the pump–probe spectra of the uncoordinated ACA isomers, see Figure 3a–c. Nanosecond measurements (1–100000 ns) were sufficient to capture the full excited state dynamics for the uncoordinated ACA isomers. For all three uncoordinated ACA isomers, the pump–probe spectra show two broad S_*n*_ ← S_1_ PIAs: one below 450 nm and one between 480–700 nm, both visible at early delay times, along with the previously described T_*n*_ ← T_1_ PIA that appears at later times. The S_*n*_ ← S_1_ PIA and T_*n*_ ← T_1_ PIA directly track the S_1_ and T_1_ population, respectively. We decomposed the kinetics of the S_*n*_ ← S_1_ PIAs and T_*n*_ ← T_1_ PIA using a genetic algorithm (see Supporting Information for details). For each of the ACA isomers, we extracted the triplet formation time from the rise of the T_*n*_ ← T_1_ PIA. This rise was found to be concomitant with the decay of the S_*n*_ ← S_1_ PIAs, suggesting that triplet formation occurs via ISC. The triplet formation times, i.e., ISC rates, were determined to be 10.3 ns (9.7 × 10^7^ s^–1^, 1-ACA), 16.7 ns (6.0 × 10^7^ s^–1^, 2-ACA), and 11.5 ns (8.7 × 10^7^ s^–1^, 9-ACA).

**Figure 3:**
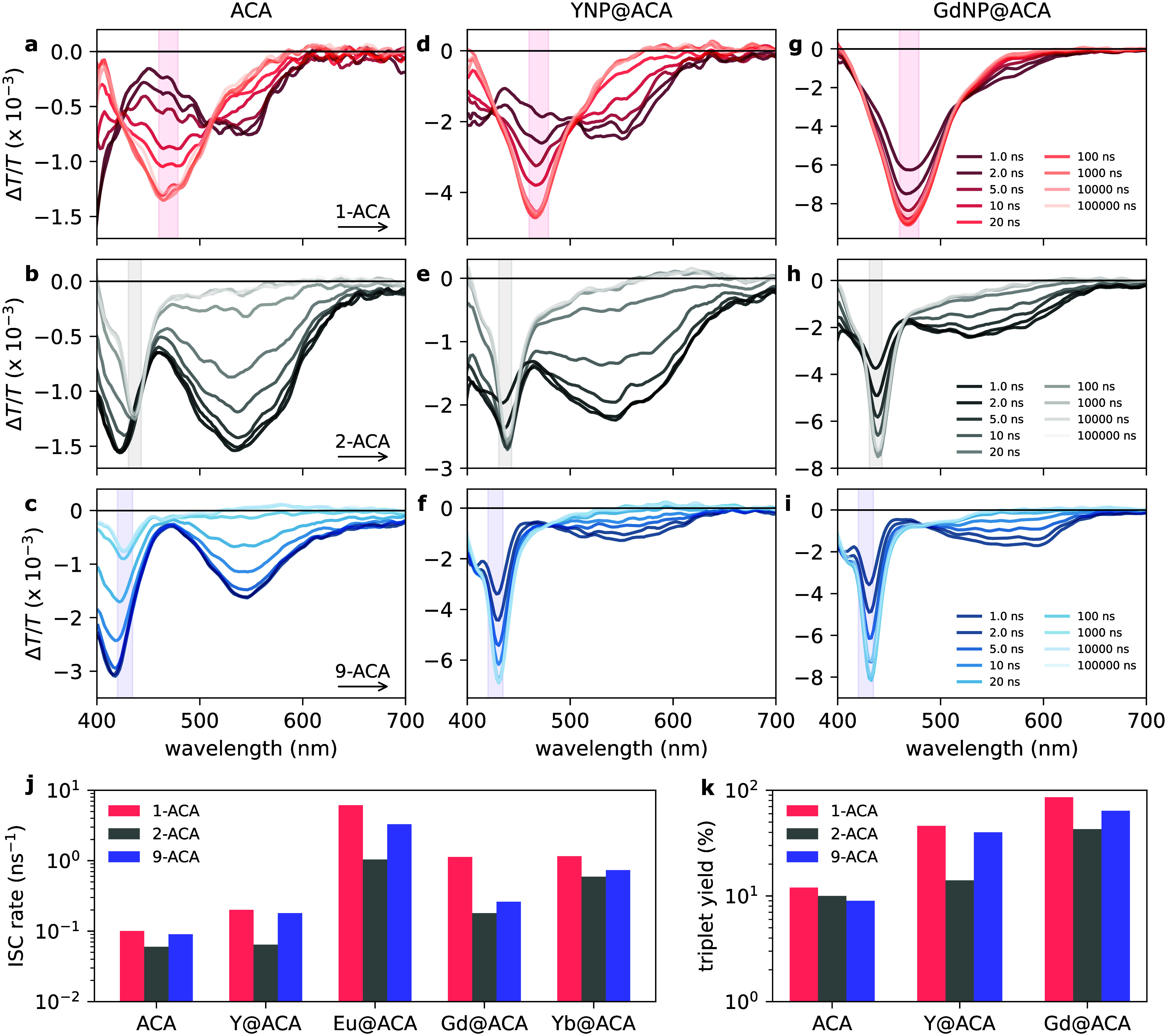
Excited state dynamics of uncoordinated ACA isomers (a–c), YNP@ACA nanohybrids (d–f), and GdNP@ACA nanohybrids (g–i) measured through pump–probe spectroscopy under 355 nm excitation at a fluence of 50 μJ/cm^2^. The legend applies to all spectra shown in that specific row. The shaded region in each plot corresponds to the ACA T_*n*_ ← T_1_ PIA as determined from triplet sensitization experiments (see Figure S9). (j) Summary of the fitted ISC rates from kinetic modeling of the pump–probe data for uncoordinated ACA isomers and LnNP@ACA nanohybrids. (k) Summary of the calculated triplet exciton yields in uncoordinated ACA isomers and YNP@ACA and GdNP@ACA nanohybrids.

Based on the triplet extinction coefficients and the maximum Δ*T*/*T* signal at the peak of the T_*n*_ ← T_1_ PIA, we calculated the triplet yields to be 12% for 1-ACA, 10% for 2-ACA, and 9% for 9-ACA. In the absence of oxygen, no significant decay of the T_*n*_ ← T_1_ PIA was observed within 0.1 ms, our maximum pump–probe delay time. This is consistent with the long triplet lifetimes typically reported for anthracene derivatives.^3^

To understand the low triplet exciton yields in the uncoordinated ACA isomers, we performed quantum chemical calculations using time-dependent density functional theory (TD-DFT) to gain deeper insight into their excited electronic states. For all isomers, the first two excited singlet states and the first four excited triplet states were found to exhibit ππ* character. Some of the higher lying singlet excited states displayed nπ* character, but these transitions occurred at wavelengths well below 300 nm. Figure S6 shows that for all ACA isomers, the T_2_ states are almost isoenergetic with the S_1_ states, although in all cases, the T_2_ states are higher in energy than the S_1_ states. To investigate the likelihood of ISC in these systems, we computed the SOC constants between the S_1_ state and the first three excited triplet states at the Franck–Condon geometry (Table S3). While the SOC constants are highest between S_1_ and T_2_, they are below 0.80 cm^–1^ for all three isomers, thereby explaining the low triplet yields in the uncoordinated isomers.

### Triplet Exciton Generation Rates and Yields in LnNP@ACA Nanohybrids

Next, we investigated the triplet exciton dynamics in the LnNP@ACA nanohybrids. Figure 3a-i show the nanosecond pump–probe spectra of the uncoordinated ACA isomers and the YNP@ACA and GdNP@ACA nanohybrids, in which no energy transfer can occur. A full overview of all pump–probe spectra and kinetics of the T_*n*_ ← T_1_ PIAs can be found in Figures S10–S15. Similarly to the uncoordinated ACA isomers, the pump–probe spectra of the LnNP@ACA nanohybrids show two broad S_*n*_ ← S_1_ PIAs between 350–420 nm and 500–700 nm, as well as the T_*n*_ ← T_1_ PIA of the ACA isomers. As for the uncoordinated isomers, the rise time of the T_*n*_ ← T_1_ PIA and decay time of the S_*n*_ ← S_1_ PIAs were found to be concomitant, indicating triplet formation via ISC.

From the signal intensity of the T_*n*_ ← T_1_ PIA and the rate of appearance of this PIA, it is evident that the excited state dynamics are accelerated upon coordination of the ACA isomers onto the LnNPs. Using the obtained T_*n*_ ← T_1_ PIA extinction coefficients, we calculated the maximum triplet yields in the YNP@ACA and GdNP@ACA nanohybrids from the peak of the T_*n*_ ← T_1_ PIAs (see Figure 3k). We find a clear increase in triplet yields following ACA coordination from 9–12% for uncoordinated ACA isomers, to 14–46% for YNP@ACA nanohybrids, and 43–86% for GdNP@ACA nanohybrids. We consistently find triplet exciton yields to be highest for 1-ACA and lowest for 2-ACA. As TET depletes the triplet exciton population in EuNP@ACA and YbNP@ACA nanohybrids and as BET can generate triplet excitons in EuNP@ACA systems too, we only compare triplet exciton yields between uncoordinated ACA isomers and YNP@ACA and GdNP@ACA nanohybrids.

Having successfully enhanced triplet exciton generation yields in the LnNP@ACA nanohybrids, we developed a kinetic model (Figure S16) to fit the excited state dynamics and extract the ISC rates for all LnNP@ACA nanohybrids. The Jablonski diagram shown in Figure 4 illustrates the modeled states and processes involved. Since we also have access to the dynamics of the S_1_ population from the S_*n*_ ← S_1_ PIAs, we include this information to make our model more robust. The need for a kinetic model and its details are outlined in the Supporting Information. Four coupled differential rate equations were used to describe the system and the obtained triplet yields were used to scale the kinetics that track the S_1_ and T_1_ populations. The experimental data and the corresponding fits using the kinetic model are shown in Figures S17–S19. A summary of the fitted parameters is presented in Table 1.

**Figure 4:**
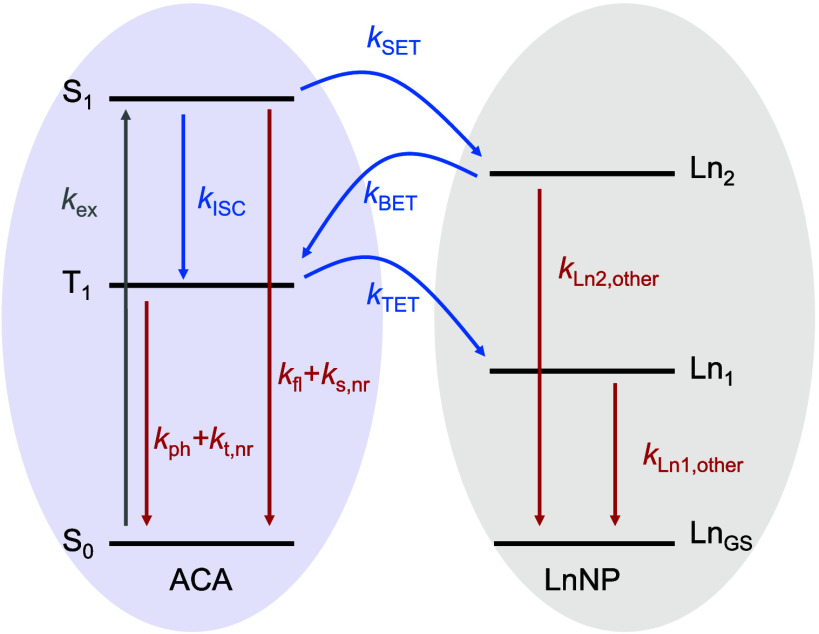
Jablonski diagram illustrating the excited states used in the kinetic model to extract the rates of ISC, TET, BET, and SET. A full description of the involved states, rate constants and the coupled differential equations used to describe the system can be found in the Supporting Information.

**Table 1:** Summary of the Triplet Exciton Yields, Triplet Exciton Lifetimes, ISC Rates, TET Rates, BET Rates, and SET Rates for All ACA Isomers and LnNP@ACA Nanohybrids

system	triplet yield^a^ (%)	triplet lifetime	*k*_ISC_ (× 10^8^ s^–1^)	*k*_TET_ (s^–1^)	*k*_BET_ (× 10^7^ s^–1^)	*k*_SET_ (× 10^8^ s^–1^)
1-ACA	12	≫1 ms	0.97	-	-	-
2-ACA	10	≫1 ms	0.60	-	-	-
9-ACA	9	≫1 ms	0.87	-	-	-
YNP@1-ACA	46	≫1 ms	1.97	-	-	-
YNP@2-ACA	14	≫1 ms	0.64	-	-	-
YNP@9-ACA	40	≫1 ms	1.82	-	-	-
GdNP@1-ACA	86	1.2 ms	11.3	-	-	-
GdNP@2-ACA	43	1.3 ms	1.83	-	-	-
GdNP@9-ACA	66	0.4 ms	2.55	-	-	-
EuNP@1-ACA	-	1.59 μs^b^	61.3	6.30 × 10^5^	28.0	58.0
EuNP@2-ACA	-	6.58 μs^b^	10.4	1.52 × 10^5^	8.02	3.9
EuNP@9-ACA	-	0.29 μs^b^	32.9	34.3 × 10^5^	9.87	44.9
YbNP@1-ACA	-	36.0 ns^b^	11.4	2.78 × 10^7^	-	2.65
YbNP@2-ACA	-	74.1 ns^b^	5.91	1.35 × 10^7^	-	1.03
YbNP@9-ACA	-	9.01 ns^b^	7.31	11.1 × 10^7^	-	2.13

a For EuNP@ACA and YbNP@ACA nanohybrids, the rise and decay of the T_*n*_ ← T_1_ PIA are convoluted due to TET occurring. Additionally, in EuNP@ACA nanohybrids, triplet formation also proceeds via BET. As a result, the triplet yields of these systems are not directly comparable and are not included.

b The triplet lifetimes for EuNP@ACA and YbNP@ACA nanohybrids are calculated as the inverse of the TET rate. As other triplet deactivation pathways are much slower, TET can be assumed to be primarily responsible for deactivating the triplet excitons in these systems.

For the uncoordinated ACA isomers, we find that ISC rates are highest for 1-ACA and 9-ACA, and are approximately 1.5 times slower for 2-ACA. Upon coordination of the ACA isomers to the LnNPs, all ISC rates are found to increase. We observe similar trends among the three isomers for all LnNPs, where ISC rates are consistently fastest for 1-ACA, followed by 9-ACA, and slowest for 2-ACA, as shown in Figure 3j. For YNPs and YbNPs, the relative ratio of the ISC rates between the ACA isomers remains similar from the uncoordinated ACA isomers. For EuNPs and GdNPs, we observe significantly larger differences between ISC rates, with rates for 1-ACA being an order of magnitude faster than those for 2-ACA: i.e., an ISC rate of 1.1 × 10^9^ s^–1^ is found for GdNP@1-ACA versus 1.8 × 10^8^ s^–1^ for GdNP@2-ACA, underscoring the importance of molecular orientation of OSCs at organic–inorganic interfaces to facilitate efficient triplet exciton generation.

### Triplet Exciton Lifetimes in YNP@ACA and GdNP@ACA Nanohybrids

We next investigated the triplet exciton lifetimes in the absence of TET, specifically in the presence of YNPs and GdNPs, which lack energy levels capable of accepting energy from the ACA isomers. For uncoordinated ACA isomers, we do not see any decay of the T_*n*_ ← T_1_ PIA over our maximum pump–probe delay of 0.1 ms.

As discussed in the previous section, coordination of the ACA isomers onto YNPs and GdNPs enhances triplet exciton generation by accelerating the S_1_ → T_*n*_ ISC process. One might therefore expect ISC from the T_1_ state back to the singlet ground state to be accelerated as well, leading to shorter triplet lifetimes. In GdNP@ACA nanohybrids, we observe a modest reduction in triplet lifetime compared to the uncoordinated molecules. Even so, because of the long intrinsic millisecond ACA triplet lifetimes, the lifetimes remain long in YNP@ACA and GdNP@ACA nanohybrids: no decay of the T_*n*_ ← T_1_ PIA was detected for YNP@ACA nanohybrids within our measurement window, and for GdNP@ACA nanohybrids the fitted monoexponential decays yielded triplet exciton lifetimes of 1.2 ms (1-ACA), 1.3 ms (2-ACA), and 0.4 ms (9-ACA). These results show that triplet yields can be dramatically increased, from 12% for uncoordinated 1-ACA to 86% for 1-ACA coordinated to GdNPs, while maintaining triplet lifetimes in the millisecond regime.

### Triplet Energy Transfer Rates in EuNP@ACA and YbNP@ACA Nanohybrids

Next, we investigated the TET dynamics in EuNP@ACA and YbNP@ACA nanohybrids. In the case of EuNPs, TET occurs to the nonemissive ^7^F_J_ manifold, while for YbNPs, TET occurs to the emissive ^2^F_5*/*2_ level. Figures 5a and 5b show the absorption of the EuNPs and YbNPs, together with the 9-ACA fluorescence and phosphorescence. When coordinated onto EuNPs or YbNPs, we attribute the accelerated decay of the T_*n*_ ← T_1_ PIA of the ACA isomers to TET. The normalized kinetic traces extracted from these PIAs are shown in Figures 5c and 5d. No spectral signatures corresponding to the ACA radical anion or cation were observed.^42,43^ Furthermore, the triplet exciton dynamics showed no dependence on solvent polarity. This allows us to rule out electron or hole transfer mediated TET, consistent with the concerted Dexter-type mechanism we reported previously.^32^

**Figure 5:**
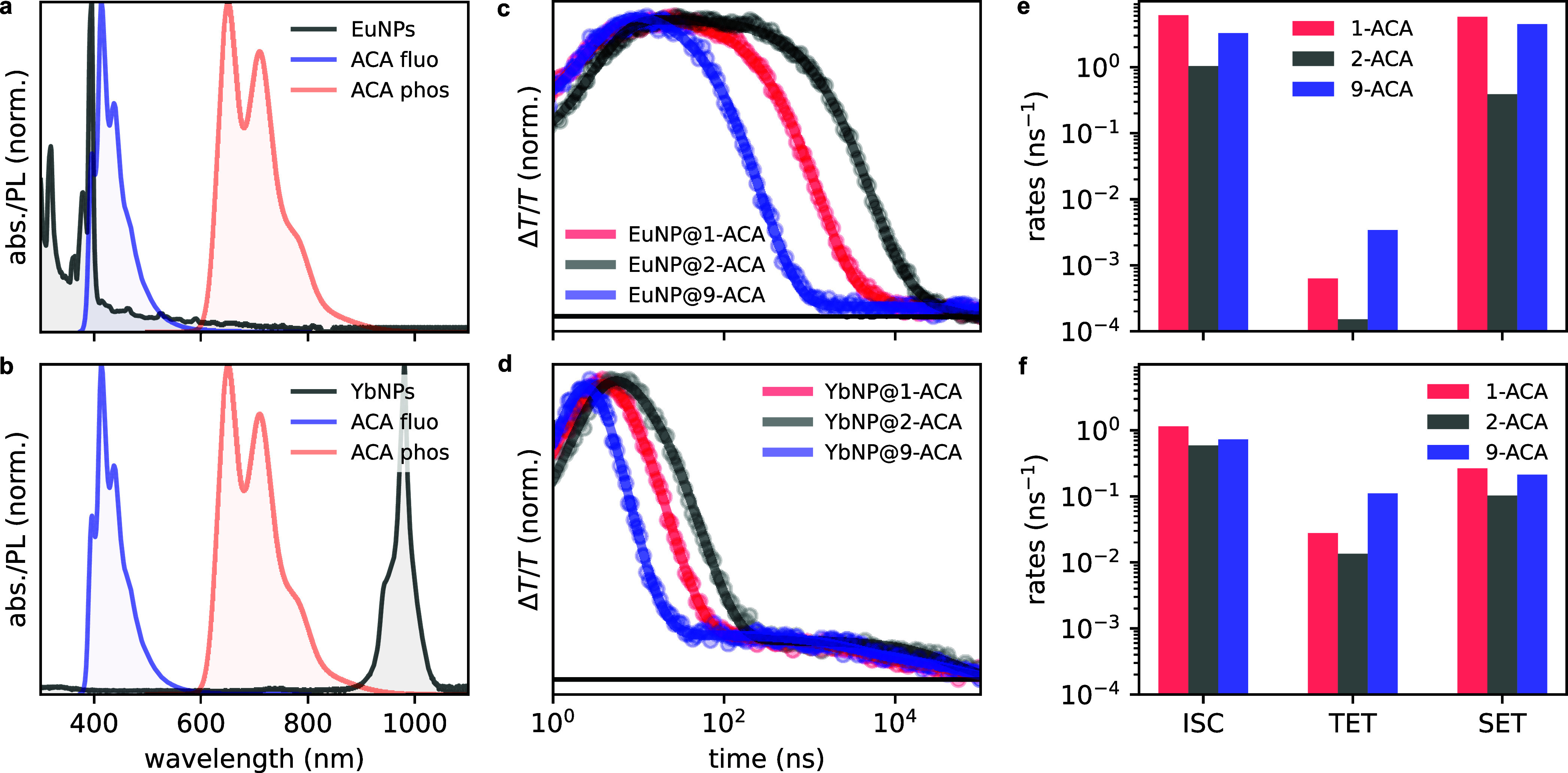
(a, b) Absorption spectra of EuNPs (black, a) and YbNPs (black, b) and PL spectra of 9-ACA: fluorescence (blue) and phosphorescence (orange). (c, d) Normalized kinetics extracted from the T_*n*_ ← T_1_ PIA of the EuNP@ACA (c) nanohybrids and YbNP@ACA (d) nanohybrids (dots) with superimposed fittings (lines), measured through pump–probe spectroscopy under 355 nm excitation at a fluence of 50 μJ/cm^2^. (e, f) Summary of the fitted ISC, TET, and SET rates of the three EuNP@ACA (e) nanohybrids and YbNP@ACA (f) nanohybrids from kinetic modeling.

We extracted the TET rates (see Figures 5e and 5f) from the kinetic model presented in the Supporting Information. We previously showed preferential binding of three anthracene derivatives to the Ln^3+^ ions on the nanoparticle surface rather than to the Na^+^ ions.^32^ This ensures direct coordination and therefore close coupling between the energy-donating ACA ligands and energy-accepting Ln^3+^ ions. Similar trends were observed for both EuNPs and YbNPs, with TET rates being highest for 9-ACA, followed by 1-ACA, and lowest for 2-ACA. The TET rate in EuNP@9-ACA was found to be over 20 times faster than in EuNP@2-ACA. This is in good agreement with previous papers that indirectly investigated TET through upconversion experiments.^25^ Since the energy levels of the three ACA isomers are almost identical, this difference is most likely a direct consequence of the wave function overlap required for a through-bond Dexter-type process. We also studied the TET rates indirectly in YbNP@ACA nanohybrids by measuring the oxygen sensitivity of the Yb­(III) emission, as reported in the Supporting Information (Figure S4).

The quantum efficiencies of the TET process were calculated to be 98.4% (1-ACA), 93.4% (2-ACA), and 99.7% (9-ACA) in EuNP@ACA, and 99.9% for all ACA isomers in YbNP@ACA, using a lower bound of 0.1 ms for the triplet exciton lifetime in the absence of energy transfer pathways. These results show that photogenerated triplet excitons on the ACA isomers can be transferred over a wide range of time scales with near-unity efficiencies to the EuNPs and YbNPs. We note that in the presence of YbNPs, the T_*n*_ ← T_1_ PIA kinetics show two clearly distinguishable decay regimes. We return to this in the Discussion section.

### Singlet and Back Energy Transfer Rates in EuNP@ACA and YbNP@ACA Nanohybrids

As pump–probe measurements provided us with insights into the excited state dynamics of the S_1_ population too, we used the extracted kinetics from the S_*n*_ ← S_1_ PIAs together with our kinetic model to determine the SET rates in EuNP@ACA and YbNP@ACA too (see Table 1). We observe similar trends to those found for ISC rates, with 1-ACA exhibiting the fastest SET rates, followed by 9-ACA and 2-ACA. The SET rate for EuNP@1-ACA was found to be 15 times faster than that of EuNP@2-ACA. SET is expected to occur via a through-space Förster resonance energy transfer process.^32,44,45^ This implies the through-space coupling in these systems is highest for 1-ACA and lowest for 2-ACA. BET rates for EuNP@ACA nanohybrids are reported too. BET in these systems populates the ACA T_1_ states and explains the high triplet exciton yields in EuNP@ACA nanohybrids, where triplet exciton formation can occur via both direct ISC and via BET from the Eu­(III) ^5^D_0_ level to the ACA T_1_ states.

## Discussion

We used pump–probe spectroscopy to demonstrate how the triplet exciton dynamics in LnNP@OSC nanohybrids are affected by molecular substitution in three ACA isomers. In uncoordinated ACA isomers, we observed negligible effects of isomeric carboxylic acid substitution on the triplet exciton dynamics. All three isomers exhibited low triplet exciton yields (∼10%), attributed to low SOC constants between the S_1_ and T_2_/T_1_ states, and long triplet lifetimes well beyond 0.1 ms.

Upon coordination onto the LnNPs, however, clear differences in triplet exciton dynamics emerged. We consistently observed the fastest triplet exciton generation for 1-ACA and the slowest for 2-ACA. While changes in the dielectric environment may influence the excited-state energy levels of the isomers and modulate ISC rates, such effects are expected to be comparable across all isomers and LnNPs, and thus cannot account for the observed differences.

We suggest that through-space interactions between the ACA ligands and the LnNP surface could play a significant role in facilitating ISC. To examine this, we consider the molecular dimensions of the ACA isomers and the structural parameters of the host matrix. The ACA anthracene core has an average length of 1.18 nm, while the average spacing between lanthanide ions in a NaLnF_4_ crystal is approximately 0.65 nm for Gd­(III). Figure S20 illustrates the suspected orientation of the ACA isomers on the LnNP surface and highlights the enhanced potential of 1-ACA to interact with multiple lanthanide ions.

Additionally, we consider that a key structural factor influencing the ISC rates is the spatial arrangement of the relevant excited state wave functions relative to the coordinated lanthanide ions, which plays a dominant role in modulating the electronic coupling. TD-DFT calculations (Figure S6 and Table S2) reveal that these wave functions in 1-ACA tail more prominently into the carboxylic acid linker than in 2-ACA and 9-ACA. Differences in ISC rates could thus partially be caused by the spatial extension of the excited state wave functions into the carboxylic acid linking group. We consider these factors together explain the more rapid ISC rates for 1-ACA than 9-ACA and 2-ACA.

While the goal of this study is to investigate differences in triplet exciton dynamics between the ACA isomers, we observed notable differences in ISC rates between the various LnNPs. Significantly faster ISC rates were observed in the presence of EuNPs, where ISC rates were found to be over 60 times larger than for uncoordinated ACA isomers, i.e., an ISC rate of 9.7 × 10^7^ s^–1^ is found for uncoordinated 1-ACA, 2.0 × 10^8^ s^–1^ for YNP@1-ACA, 6.1 × 10^9^ s^–1^ for EuNP@1-ACA, 1.1 × 10^9^ s^–1^ for GdNP@1-ACA, and 1.1 × 10^9^ s^–1^ for YbNP@1-ACA. This suggests that direct SOC, the strength of which is expected to increase with increasing atomic number, is not solely responsible for triplet exciton generation. Previous studies have suggested that spin-exchange coupling interactions with unpaired lanthanide 4f electrons could result in accelerated rates of ISC, which can explain the difference in ISC rates between the diamagnetic YNPs and the paramagnetic EuNPs, GdNPs, and YbNPs.^4,46^ Additionally, the magnetic moments of the coordinated lanthanide ions could affect and be responsible for the observed ISC accelerations.^47^ Lastly, the observation that ISC rates are highest in the presence of EuNPs and YbNPs could indicate that electronic coupling is (partially) responsible for the observed accelerations too. Disentangling these effects would ideally require examination of the entire lanthanide series and is beyond the scope of the current work.

TET was found to be consistently fastest for 9-ACA and slowest for 2-ACA, consistent with a Dexter-type through-bond energy transfer mechanism.^32^ Here, the spatial overlap of the T_1_ wave function with 4f acceptor orbitals of the Ln­(III) ions is critical. TD-DFT calculations (Figure S6 and Table S2) show excited state planarization between the anthracene core and carboxylic linking group. In the excited state, reduction of the dihedral angle increases π-conjugation and facilitates greater wave function leakage onto the carboxylic acid group. In 9-ACA in particular, this enhances delocalization of the T_1_ wave function, maximizing overlap with the Ln­(III) ion 4f surface orbitals, decreasing the through-bond distance, and resulting in the fastest TET rates.

Notably, TET rates were 2 orders of magnitude faster in YbNP@ACA than EuNP@ACA nanohybrids. Future work will investigate the effect of the lanthanide ion in more detail. In the presence of YbNPs, we observe that the T_*n*_ ← T_1_ PIA kinetics do not decay to zero (see Figure 5d), suggesting either incomplete TET or heterogeneous dynamics. While incomplete TET seems unlikely given the high ratio of Yb­(III) acceptor ions to ACA isomers per LnNP, we propose that a dynamic equilibrium exists between coordinated and uncoordinated ACA isomers. Coordinated ligands undergo rapid TET, while uncoordinated ones rely on diffusion to the LnNP surface before TET can occur. The longer-lived triplet exciton population showed no fluence dependence, confirming that this spectral feature does not originate from multiexciton effects.

## Conclusion

In conclusion, we show that by carefully engineering the coordination geometries of OSCs and the wave function overlap between OSCs and LnNPs, triplet exciton generation and transfer can be accelerated by 1–2 orders of magnitude through relatively small changes in molecular structure. Interestingly, triplet lifetimes remain well beyond 0.1 ms for all LnNP@ACA nanohybrids in the absence of TET. This makes these systems highly attractive for applications that benefit from long-lived triplets, such as photocatalysis. In comparison to previous studies that exclusively investigated TET, we present a comprehensive picture of the triplet exciton dynamics, including triplet exciton yields, ISC rates, triplet lifetimes, TET rates, and TET efficiencies. In doing so, we establish structure–function relationships and a detailed molecular-level understanding of the triplet exciton dynamics across the hybrid organic–inorganic interface in LnNP@OSC systems. By linking molecular design to material performance, this work paves the way for future studies aimed at optimizing hybrid systems for energy conversion applications.

## Supplementary Material



## Data Availability

The data that support the findings of this study are openly available in Apollo – University of Cambridge Repository at https://doi.org/10.17863/CAM.121253.

## Notes

Experimental details, materials and methods, and additional supporting experimental data including TEM images, DFT calculations, ligand coverage calculations, photoluminescence spectra, PLQE measurements, further picosecond and nanosecond transient absorption, nanosecond transient photoluminescence, triplet sensitization data, triplet yield calculations, and a description of the kinetic models (PDF)
