# Supplementary material for: Molecular Orientation Controls Triplet Exciton Dynamics in Organic Molecules Coupled to Lanthanide-Doped Nanoparticles
Source: J Am Chem Soc. 2025 Oct 2;147(41):37788–97. doi: 10.1021/jacs.5c13962 (PMC12532298; doi:10.1021/jacs.5c13962)
Supplement: Supplementary file 1 [file ja5c13962_si_001.pdf]

Molecular Orientation Controls Triplet Exciton Dynamics in Organic Molecules Coupled to Lanthanide-Doped Nanoparticles

Lars van Turnhout,[1] Alasdair Tew,[1] Simon A. Dowland,[1] Ebin Sebastian,[1] Zhao Jiang,[1] Rakesh Arul,[1] Zhongzheng Yu,[1] Akshay Rao[1*]

[1] Cavendish Laboratory, University of Cambridge, Cambridge, CB3 0US, United Kingdom

* E-mail address: ar525@cam.ac.uk (A. Rao).

Table of Contents

Methods and Materials 3
Materials 3
Synthesis of $NaLnF_4$ Nanoparticles (LnNPs, Ln = Y/Eu/Gd/Yb) 3
Surface Modification of LnNPs with ACA 3
Transmission Electron Microscopy 4
Optical Spectroscopy: General 4
Steady-State Absorption and Photoluminescence 4
Phosphorescence measurements 4
Time-Resolved Photoluminescence 5
Photoluminescence Quantum Efficiency (PLQE) 5
Femtosecond Transient Absorption 5
Nanosecond Transient Absorption 6
Genetic Algorithm 6
Kinetic Modelling 7
TEM images 8
State Diagram 9
Absorption Spectra 10
Ligand coverage of LnNPs 11
Emission Spectra 13
Time-Resolved Photoluminescence Spectra 15
PLQE Measurements 16
DFT Calculations 17
Energy Level Diagrams 17
Spin-Orbit Coupling Constants 20
Triplet Sensitisation Measurements 21
Triplet Yield Calculations 21
Further Transient Absorption Spectroscopy 24
Kinetic Modelling 30
Orientation of ACA Isomers 36
References 37

Methods and Materials

Materials

Yttrium acetate hydrate ($Y(CH_3COO)_3{\cdot}xH2O$, 99.9%), europium acetate hydrate ($Eu(CH_3COO)_3{\cdot}xH2O$, 99.9%), gadolinium acetate hydrate ($Gd(CH_3COO)_3{\cdot}xH2O$, 99.9%), ytterbium acetate hydrate ($Yb(CH_3COO)_3{\cdot}xH2O$, 99.9%), sodium hydroxide (NaOH, >98%), ammonium fluoride (NH4F, 99%), 1-octadecene (ODE, 90%), oleic acid (OA, 90%) and all anhydrous solvents (toluene, hexane, ethanol, and THF) were purchased from Sigma-Aldrich. 1-anthracenecarboxylic acid (97%) (1-ACA), 2-anthracenecarboxylic acid (98%) (2-ACA) and 9-anthracenecarboxylic acid (99%) (9-ACA) were obtained from TCI America. If not stated otherwise, all chemicals were used as received without further purification.

Synthesis of $NaLnF_4$ Nanoparticles (LnNPs, Ln = Y/Eu/Gd/Yb)

The synthesis of the lanthanide-doped nanoparticles (LnNPs) was adapted from a well-documented method by Wang et al.[1] The whole synthesis was carried out under a continuous nitrogen gas flow. In a typical experiment, $Ln(CH_3COO)_3$ (1 mmol) was mixed with 3 mL of OA and 70 mL of ODE in a 50 mL three-neck round-bottom flask. The nanoparticle precursor was obtained by heating the mixture to 140 °C for 1 h under continuous magnetic stirring. Thereafter, the solution was cooled back to 70 °C. A methanol solution (7 mL) containing NH_4F (0.96 mmol) and NaOH (0.6 mmol) was added and the solution was stirred at 70 °C for 45 min to remove any residual methanol. The reaction temperature was then raised to 300 °C and the reaction was maintained at this temperature for 40 min to allow the growth of nanoparticles. Following cooling to room temperature, the nanoparticles were obtained through repeated precipitation, with ethanol, and centrifugation cycles. The resulting particles were re-dispersed in toluene at a concentration of 30 mg/mL.

Surface Modification of LnNPs with ACA

Ligand exchange reactions were carried out to attach the three ACA isomers (ACA-1, ACA-2, ACA-9) onto the $NaLnF_4$ nanoparticles. In these reactions, 1 mL of the OA-capped $NaLnF_4$ nanoparticles (30 mg/mL) in toluene were mixed with various amounts of a 1 mg/mL solution of ACA in THF. The volume used for each ligand were: 0.1 mL of ACA-1, 0.2 mL of ACA-2, 0.5 mL 0f ACA-9. Different

volumes were used as the different ACA isomers exhibited different binding affinities towards the LnNPs – the volumes used ensured similar ligand coverage for each LnNP@ACA nanohybrid. The mixture was left overnight. Excess, i.e. uncoordinated, ligands were removed through two precipitation and centrifugation cycles, using ethanol as the antisolvent. Finally, the products were re-dispersed in toluene for optical measurements.

Transmission Electron Microscopy

Transmission electron microscopy to investigate LnNP size distribution morphology was performed using an FEI Tecnai F20 at 200 kV accelerating voltage. Samples were dropcasted from ~5 mg/mL LnNP solutions in toluene onto 200-mesh Cu grids (Agar AGS160).

Optical Spectroscopy: General

All optical measurements were carried out in 1 mm pathlength quartz cuvettes. Samples were prepared in a nitrogen-atmosphere glovebox using degassed, anhydrous solvents. Cuvettes were sealed prior to measurements with a PTFE cap, PTFE tape, and parafilm, to ensure the absence of oxygen inside the samples.

Steady-State Absorption and Photoluminescence

Steady-state absorption across the UV/Vis/NIR spectral regions were carried out using a Shimadzu UV3600Plus spectrometer.

Steady-state photoluminescence spectra were measured using an Edinburgh Instruments FLS1000-DD-stm spectrometer, comprised of a 450 xenon lamp for excitation and a FLS1000 double excitation and double emission monochromator. UV/Visible emissions were detected using a PMT-980 detector. NIR emissions were detected using a PMT-1700 detector.

Phosphorescence measurements

Room temperature phosphorescence spectra were measured of dropcasted films of GdNP@ACA solutions in a microscope with 633 nm continuous wave laser excitation (<1mW power on sample), with a 100X 0.9 NA objective lens, and luminescence resolved on a dispersive-grating spectrometer with a CCD camera. A notch filter was used to remove the laser excitation from the spectrum.

Time-Resolved Photoluminescence

Time-resolved photoluminescence were carried out on an Edinburgh Instruments FLS1000-DD-stm spectrometer.

The time-resolved photoluminescence of the organic molecules (~ns timescale) was measured using time correlated single photon counting (TCSPC). Samples were excited with a pulsed 375 nm EPL-375 diode laser at a 10 MHz repetition rate (60 ps pulsewidth). Pump scatter from the laser excitation was filtered out using an appropriate Thorlabs long-pass filter in the emission path. The photoluminescence was directed through an FLS1000 double emission monochromator and detected by a PMT-980 detector in TCSPC mode. The instrument response was determined using light scattered off a piece of scratched glass at the excitation wavelength, giving a value of 137 ps.

The time-resolved photoluminescence from the lanthanide(III) ions (~µs-ms timescale) was measured in multi-channel scaling (MCS) mode in order to reduce the otherwise long measurement time if equivalent measurements were performed using the TCSPC method. Excitation was performed using a pulsed 375 nm EPL-375 diode laser to excite the ACA isomers or using a 60W xenon microsecond flashlamp at 100 Hz repetition rate (2 µs pulsewidth) for direct excitation of the lanthanide(III) ions. Detection was carried out using a PMT-980 (visible) or PMT-1700 (NIR) detector in MCS mode.

Photoluminescence Quantum Efficiency (PLQE)

PLQE measurements were carried out inside a Spectralon coated integrating sphere following the procedure of de Mello *et al.*[2] Measurements were taken at room temperature using a temperature and current controlled 375 nm Thorlabs continious wave laser diode as the excitation source. Light from the experiment was collected using an optical fibre connected to an Andor Kymera 328i Spectrometer housing a DU420A Silicon CCD detector. Setup calibration was performed using a Bentham 610 QTH calibration source. PLQE calculations were performed as described by de Mello et al.

Femtosecond Transient Absorption

The output of a titanium:sapphire amplifier system (Spectra Physics Solstice Ace) operating at 1 kHz and generating ~100 fs pulses was split into the pump and probe beam paths. The 400 nm pump pulses

were created by sending the 800 nm fundamental beam of the Solstice Ace through a SHG BBO crystal of 1 mm thickness (Eksma Optics). The pump was blocked by a chopper wheel rotating at 500 Hz to omit every second pump pulse. The ultraviolet-visible broadband probe beam was generated by focusing the 800 nm fundamental beam onto a moving CaF2 crystal (Eksma Optics, 5 mm) connected to a digital motion controller (Mercury C-863 DC Motor Controller) after passing through a mechanical delay stage (Thorlabs DDS300-E/M). Time delays of 100 fs to 2 ns were generated with the delay stage. The transmitted pulses were collected with a monochrome line scan camera (JAI SW-4000M-PMCL; spectrograph, Andor Shamrock SR-163).

Nanosecond Transient Absorption

A 355 nm (ACA excitation) or 532 nm (PdOEP excitation in triplet sensitisation experiments) pump beam were generated by the third and second harmonic, respectively, of a Q-switched Nd:YVO4 (1 ns pump length, Advanced Optical Technologies Ltd AOT-YVO-25QSPX) laser. A LEUKOS Disco 1 UV low timing jitter supercontinuum laser (STM-1-UV) was used to generate the probe beam. The probe beam was split by a 50% beam splitter into a reference and probe and both were focused and directed through the sample – only the probe beam interacted, i.e. was overlapped, with the pump beam. This allows for correction of any shot-to-shot fluctuations. A pair of line image sensors (Hamamatsu, G11608) mounted on a spectrograph (Andor Solis, Shamrock SR-303i) were used to detect the signal, using a custom-built board from Entwickslungsbüro Stresing to read out the signal. The delay was controlled electronically for all nanosecond transient absorption measurements and every second pump pulse is omitted. The average fractional differential transmission ($\Delta T/T$) of the probe is calculated after each time delay following the collection of 1000 shots.

Genetic Algorithm

Where necessary, deconvolution of TA data was performing through global analysis using a previously described genetic algorithm.[3] For spectral target analysis we predefine the spectra present and find the proportion of each spectrum present at every time point via linear regression. Global fitting was chosen over other methods such as decay associated species fitting as the latter restricts one to controlling the kinetics only rather than the spectra, whereas global fitting using a genetic algorithm allows one to fix

certain spectra. As the triplet spectrum was accurately known from sensitization experiments, this method was preferred.

Kinetic Modelling

In order to fit the excited state dynamics of the OSCs following photoexcitation, a kinetic model was created to fit the singlet exciton and triplet exciton dynamics as measured through pump-probe spectroscopy. For this purpose, short (1–2000 ps) and long time TAS data (1–100 000 ns) were captured separately. The short time measurements captured the initial nanoseconds, which was often sufficient to capture the decay of the singlet exciton population but not the triplet. Long time data up to 100 μs was stitched to the short time data and was able to capture the triplet exciton population rise and, for most samples, the full decay of the triplet exciton population. A set of ordinary differential equations (ODEs) modelled the populations of singlet, triplet and lanthanide states with shared, i.e. global, rate constants. The ODEs were integrated using the SciPy package odeint in Python and a wrapper function allowed the Lmfit minimise package to perform a global fit of the rate constants via least squares minimisation. The Lmfit package was used to enable facile modification of the parameters used for the fit and also produces parameter uncertainties based on the second derivative of fit quality. A specific description of the ODEs and fit results will be provided further on.

TEM images

Figure S1 show the TEM images recorded of the as-synthesised LnNPs (Ln = Y / Eu / Gd / Yb). The particles were found to have mean diameters of 5.2 ± 0.5 nm (YNPs), 5.1 ± 0.5 nm (EuNPs), 5.0 ± 0.4 nm (GdNPs), and 4.8 ± 0.4 nm (YbNPs).

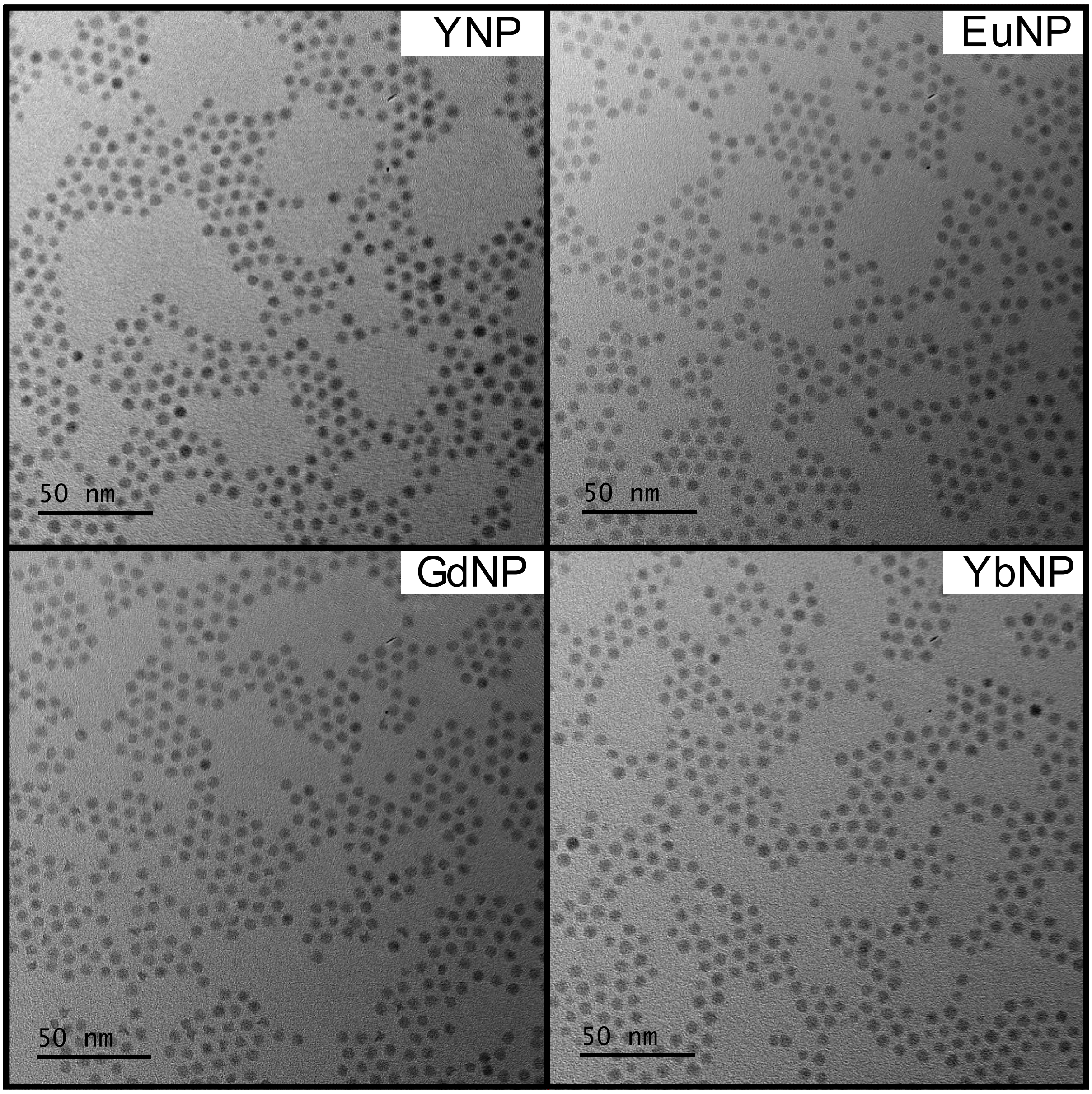

Figure S1. TEM images of the as-synthesised YNPs, EuNPs, GdNPs, and YbNPs, all with OA as the surface ligands. The scale bar is 50 nm in all images.

State Diagram

Below we outline the relevant energy levels for the used ACA ligands and the four LnNPs that were employed in this study:

ACA states	energy (eV)
S_0	0
S_1	3.1
T_1	1.8

YNP states	energy (eV)
^1S	0

EuNP states	energy (eV)	EuNP states	energy (eV)
$^7F_0$	0	$^7F_6$	0.612
$^7F_1$	0.046	$^5D_0$	2.14
$^7F_2$	0.129	$^5D_1$	2.36
$^7F_3$	0.234	$^5D_2$	2.67
$^7F_4$	0.355	$^5D_3$	3.02
$^7F_5$	0.485	$^5D_4$	3.43

GdNP states	energy (eV)	GdNP states	energy (eV)
$^8S_{7/2}$	0	$^6P_{5/2}$	4.06
$^6P_{7/2}$	3.98	$^6P_{3/2}$	4.13

YbNP states	energy (eV)
$^2F_{7/2}$	0
$^2F_{5/2}$	1.27

Absorption Spectra

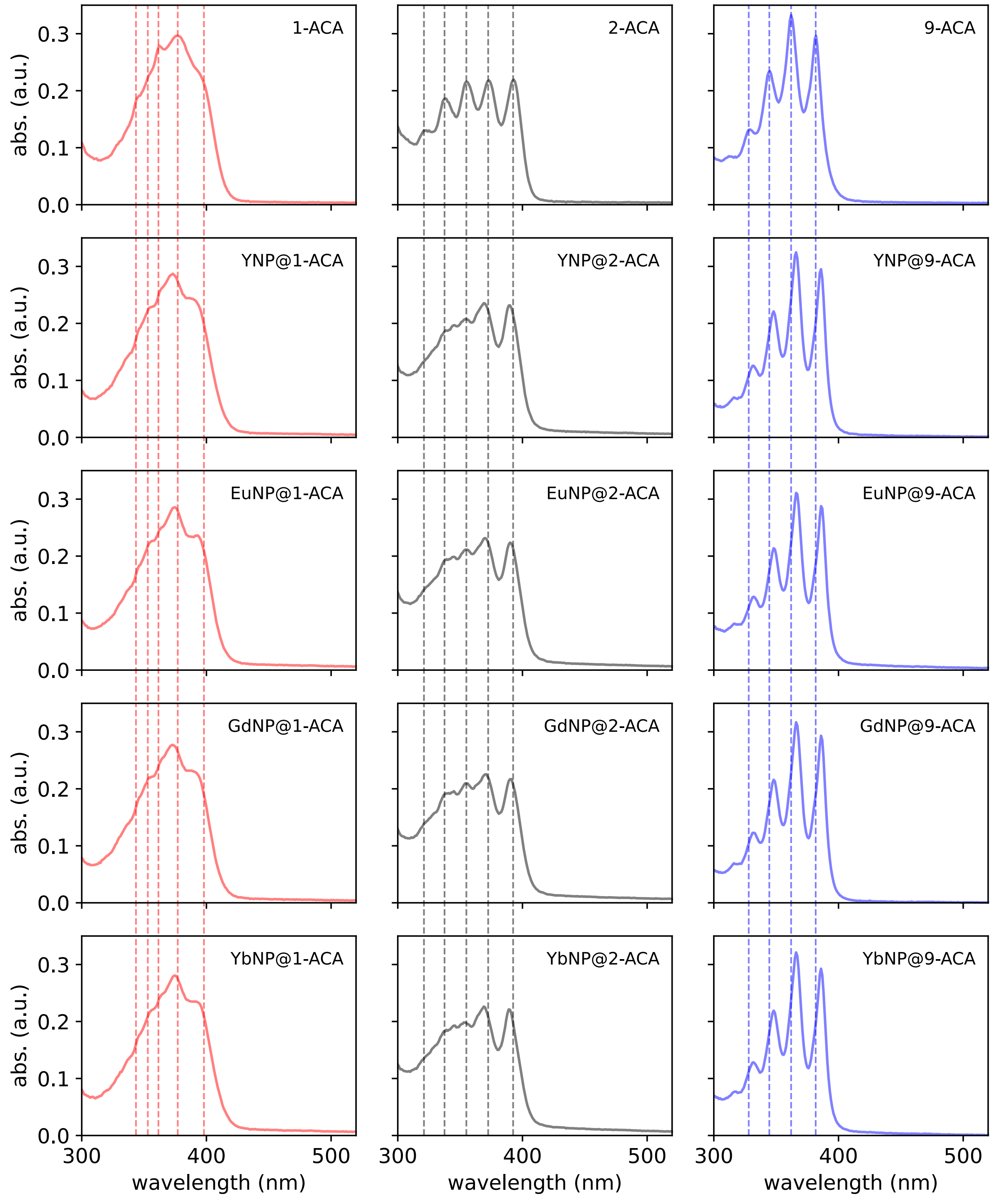

Figure S2. Steady-state absorption spectra of the uncoordinated ACA ligands and LnNP@ACA nanohybrids.

Ligand coverage of LnNPs

Ligand exchanges were carried out as described beforehand. The washed and redispersed nanohybrid systems of the type LnNP@ACA were characterised by UV-vis absorption spectroscopy to determine the ligand loading. Firstly, the molar extinction coefficients (ε) of the pristine ligands were determined by measuring the ACA absorption spectra at 7 different concentrations ranging from 0.01 mg/mL to 1 mg/mL. Linear fits of the absorbance values at each wavelength using the Beer-Lambert law

$$A = \varepsilon \times c \times l$$

yielded the extinction coefficient at each wavelength, where A is the measured absorbance, ε the molar extinction coefficient, c the concentration (in mol/L) and l the pathlength (0.1 cm). Hence ε corresponds to the slope of a plot of *A* vs *c* x *l*. In particular, the extinction coefficients at the maximum absorption wavelength were found to be 6200 M^{-1} cm^{-1} (377 nm), 5400 M^{-1} cm^{-1} (393 nm), and 8600 M^{-1} cm^{-1} (362 nm) for 1-ACA, 2-ACA, and 9-ACA, respectively. These molar extinction coefficients were used to determine the ligand coverage. For the calculation, the LnNPs were assumed to be spheres and for the sphere diameter we used to obtained average size from the TEM images.

Hence, as an example, knowing the peak absorption of GdNP@ACA-1 (see Figure 2c) and the ACA-1 extinction coefficient at the peak absorption, we can readily calculate the concentration *c* of ACA-1 ligands using the Beer-Lambert law to be 0.1 mg/mL. The amount of NPs is known to be 30 mg/mL, corresponding to an ACA-1 loading of 0.1 mg ACA-1 per 15 mg GdNPs.

Using the average TEM diameter of 5 nm (radius = 2.5 nm) of the LnNPs, we can calculate the average volume of a single NP to be

$$\frac{4}{3} \times \pi \times (2.5 \times 10^{-9})^3 \text{ m}^3$$

Assuming an average bulk density of ~ 5 g/cm^3 for the LnNPs,[4] the mass of a single nanoparticle is estimated to be 3.27 x 10^{-16} mg. Using this, we estimate the number of LnNPs in 1 mL to be ~ 4.6 x 10^{16}. Knowing an ACA concentration of 0.1 mg/mL (MW = 222 g/mol), i.e. 2.71 x 10^{17} ACA molecules per mL, we approximate the number of ACA ligands per LnNP to be ~ 6-7.

Care was taken to optimise the ligand exchange reactions such that the resulting LnNP@ACA nanohybrid had comparable ligands loadings. For example, we found ACA-1 to have a stronger binding affinity for the LnNPs than ACA-2, followed by ACA-9. Hence, different initial amounts of ACA were used during the ligand exchange process to ensure similar coverage afterwards.

Emission Spectra

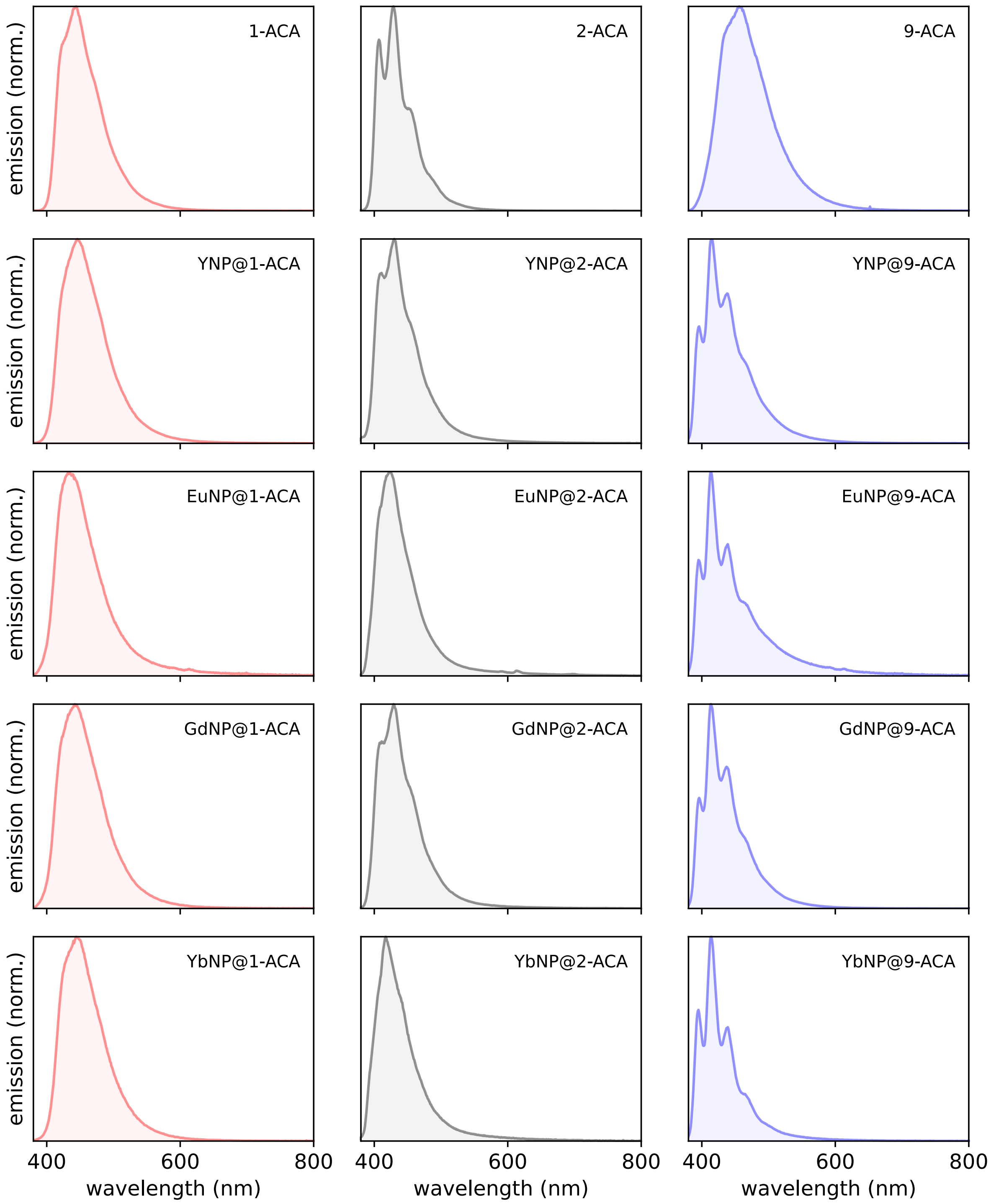

Figure S3. Steady-state emission spectra of the uncoordinated ACA ligands and LnNP@ACA nanohybrids.

As TET in YbNP@ACA nanohybrids occurs to the emissive $^2F_{5/2}$ state, we compared the Yb(III) emission in oxygen-free and aerated solutions. As shown in Figure S4, the Yb(III) emission decreases drastically in aerated solutions, presumably due to triplet ground state oxygen quenching the excited triplet states of the ACA isomers,[5] resulting in the formation of singlet oxygen.[6] Alternatively, triplet oxygen could react with excited surface Yb(III) ions; however, we consider this pathway unlikely, as we did not observe any change in the Yb(III) lifetime upon aeration of the solutions. We find that the Yb(III) emission is quenched by 62% for 1-ACA, 40% for 2-ACA, and 57% for 9-ACA.

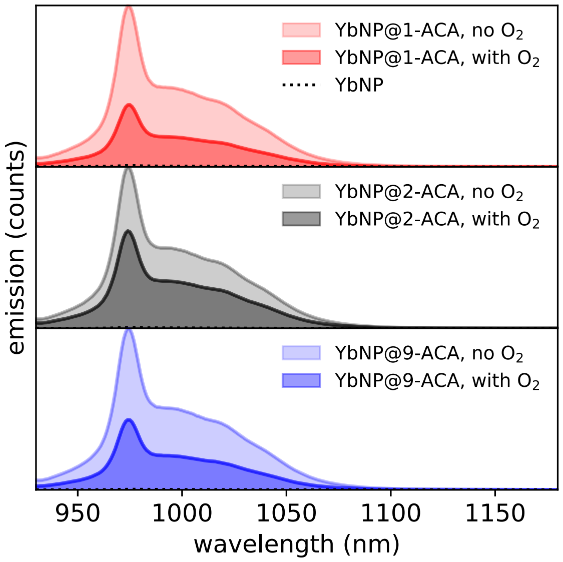

Figure S4. NIR photoluminescence spectra of the YbNP@ACA nanohybrids in the presence and absence of O_2, λ_{ex} = 355 nm.

Time-Resolved Photoluminescence Spectra

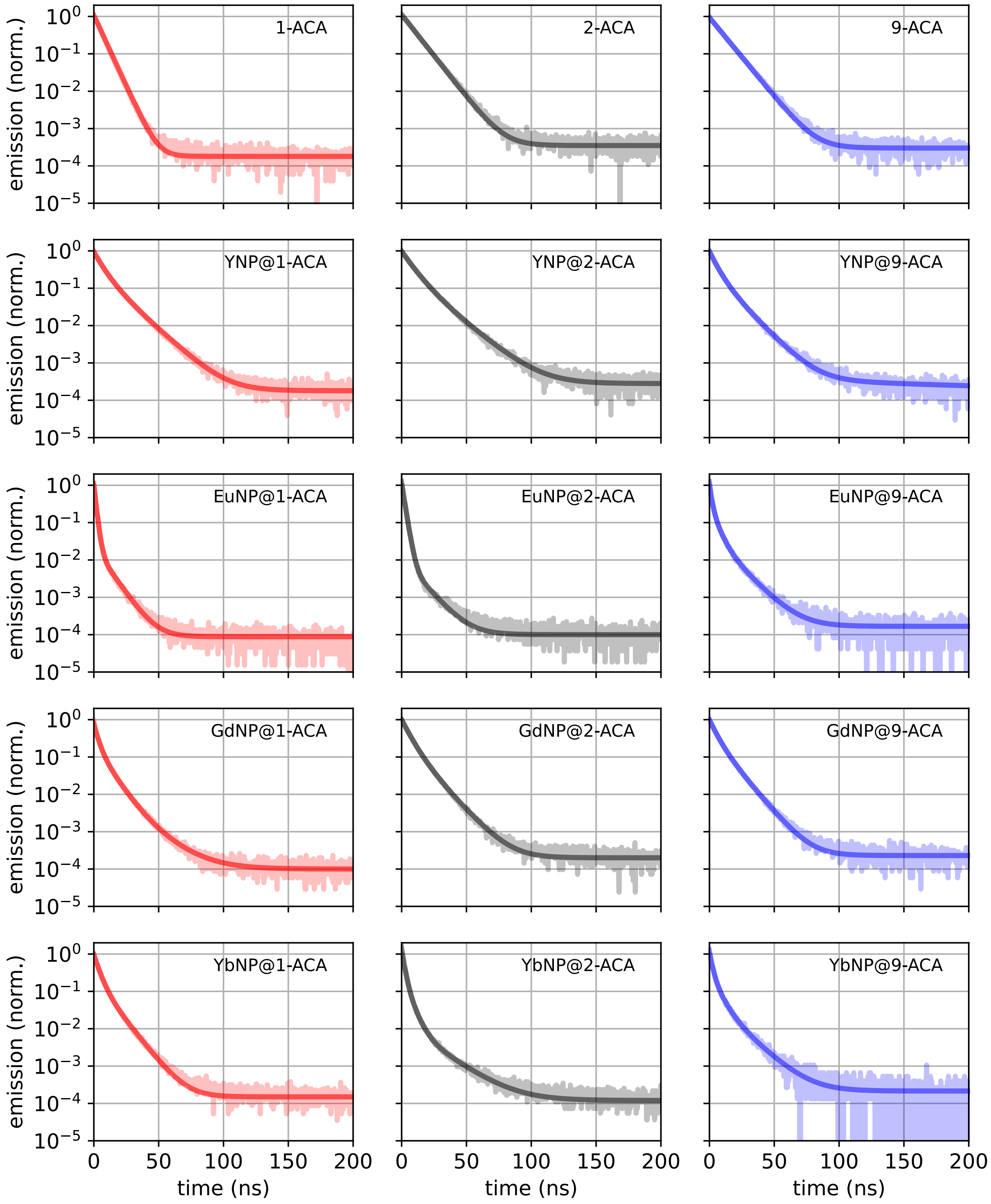

Figure S5. Time-resolved emission spectra of ACA ligands and LnNP@ACA nanohybrids.

PLQE Measurements

System	ACA PLQE (%)	$\tau_{PL,avg}$ (ns)	k_{rad} (x 10^7 s^{-1})	$\sum k_{non-rad}$ (x 10^7 s^{-1})
1-ACA	30.2	5.8	5.20	12.0
2-ACA	44.8	9.8	4.57	5.63
9-ACA	44.4	10.2	4.26	5.55
YNP@1-ACA	34.6	5.9, 13.9 (8.4)	4.1	7.8
YNP@2-ACA	30.1	7.6, 15.8 (9.9)	3.0	7.1
YNP@9-ACA	29.4	5.1, 12.2 (7.3)	3.2	10.5
GdNP@1-ACA	10.3	2.8, 8.1 (4.3)	2.4	20.8
GdNP@2-ACA	21.2	5.6, 11.8 (7.2)	2.9	10.9
GdNP@9-ACA	25.8	4.7, 10.7 (6.2)	4.2	12.0
EuNP@1-ACA	1.4	1.4, 8.7 (1.6)	0.88	61.6
EuNP@2-ACA	3.7	1.9, 10.8 (2.1)	1.76	45.9
EuNP@9-ACA	1.6	1.4, 12.5 (2.5)	0.64	39.4
YbNP@1-ACA	13.1	3.6, 9.9 (5.0)	2.6	17.4
YbNP@2-ACA	6.7	1.8, 9.1 (2.4)	2.8	38.9
YbNP@9-ACA	6.6	1.9, 9.8 (3.3)	2.0	2.83

Table S1. Visible PLQE values of uncoordinated ACA isomers and coordinated LnNP@ACA nanohybrids, observed ACA PL lifetimes from TCSPC measurements, and the calculated radiative and sum of non-radiative rate constants.

DFT Calculations

Energy Level Diagrams

Ground state geometry optimisations and harmonic oscillator frequencies of the ACA derivatives were computed using DFT with the Becke's three-parameter functional in combination with the Lee-Yang-Parr correlation functional (B3LYP) and 6-31+g(d,p) basis set in Gaussian 16. The vertical excitation energies and oscillator strengths were calculated by employing TD-DFT along with the 6-31+G(d,p) basis set and B3LYP functional. The frontier MOs were obtained from the generated cube files of the energy calculations. Hole-electron analysis generations were carried out using Multiwfn version 3.7.8.[7] Equilibrium geometries for the lowest singlet excited states were obtained at the TD-DFT/B3LYP/6-31+G(d,p) level of theory by using the ground state minima as the initial structures. A summary of the vertical excitation energies and MOs of the S_0, S_1, and T_1 states is shown in Figure S6. A summary of the dihedral angles between the anthracene core and carboxylic linking groups in the S_0, S_1, and T_1 states is presented in Table S2.

states	1-ACA	2-ACA	9-ACA
S_0	13.86	26.31	61.66
S_1	0.00	12.54	42.16
T_1	0.01	17.95	15.93

Table S2. Dihedral angles of 1-ACA, 2-ACA, and 9-ACA, computed at TDDFT/B3LYP/6-31+G(d,p)) level of theory.

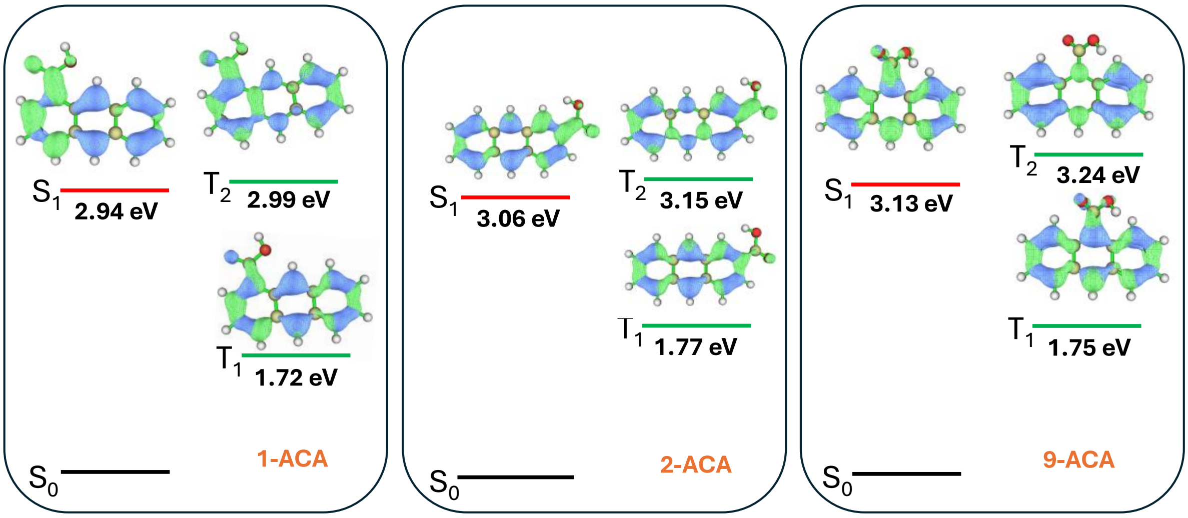

Figure S6. The energy level diagram of 1-ACA, 2-ACA, and 9-ACA showing the lowest singlet and triplet excited states calculated at the TDDFT/B3LYP/6-31+G(d,p) level of theory at the FC optimized geometry with corresponding isosurface representing the hole (blue) and electron (green) distribution.

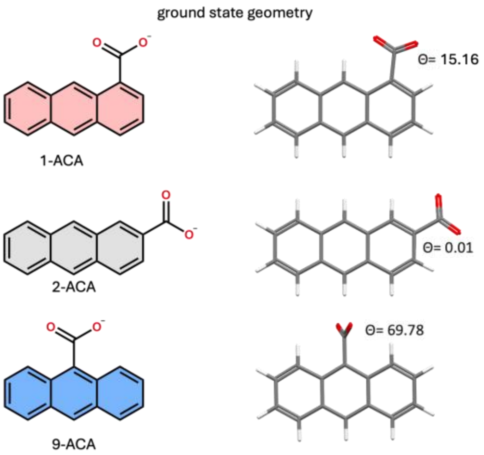

Figure S7. Molecular structures and optimized geometries of 1-ACA$^-$, 2-ACA$^-$, and 9-ACA$^-$, with the corresponding torsion angles along the carboxylic group in the ground electronic state of the deprotonated carboxylate forms of the ACA isomers.

Figure S7 shows the optimized geometries and torsion angles in the ground state of the ACA anions. These results show that deprotonation leads to modest reductions in torsional distortion, but the overall trend amongst the derivatives remains unchanged: a relatively low torsion angle is found for 1-ACA and 2-ACA, on account of reduced steric hindrance from the hydrogen atoms, and a large, near-orthogonal, torsion angle is found for 9-ACA.

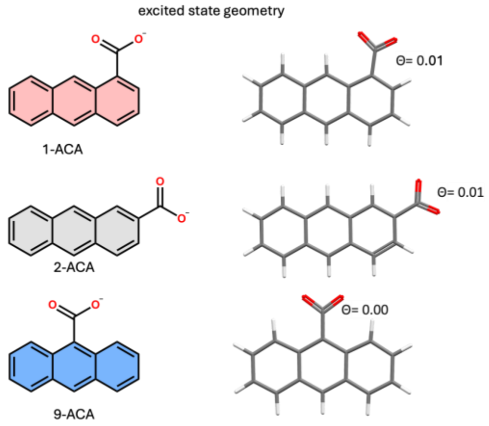

Figure S8. Molecular structures and optimized geometries of 1-ACA^{-}, 2-ACA^{-}, and 9-ACA^{-}, with the corresponding torsion angles along the carboxylic group in the excited electronic state of the deprotonated carboxylate forms of the ACA isomers.

In the excited state (Figure S8), for all three ACA isomers, we find the torsion angle for the deprotonated states to approach 0°. Especially for 9-ACA, this facilitates greater wavefunction leakage onto the carboxylic acid group, in comparison to the ground state, and could thereby reduce the effective distance between the excited state wavefunctions and the coordinated lanthanide ion, crucial for triplet energy transfer which was observed to consistently be quickest for the 9-ACA isomers.

Spin-Orbit Coupling Constants

The SOC constants at the optimised geometries were computed using the PySOC code developed by Thiel and coworkers.[8] We computed the spin-orbit coupling (SOC) constants between the S1 state and the first three excited triplet states, the values of which are shown in Table S3.

states	1-ACA	2-ACA	9-ACA
S_1/T_1	0.13	0.25	0.14
S_1/T_2	0.32	0.21	0.75
S_1/T_3	0.37	0.23	0.16

Table S3. Spin-orbit coupling constants in cm−1 between the first excited singlet and the first three excited triplet states at FC Geometries of 1-ACA, 2-ACA, and 9-ACA, computed at TDDFT/B3LYP/6-31+G(d,p)) level of theory.

Triplet Sensitisation Measurements

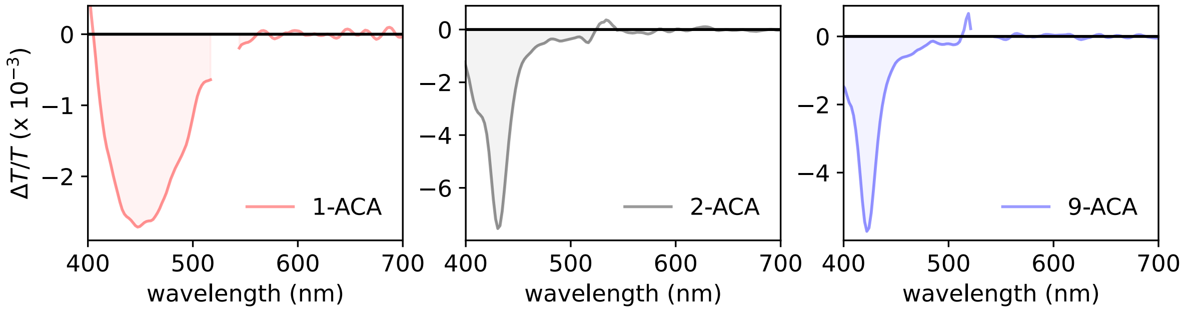

Figure S9. Triplet sensitisation measurements using PdOEP as the triplet sensitiser. The presented spectra are the spectral averages after a 20-50 μs pump-probe delay.

Triplet Yield Calculations

Triplet yields calculations were performed following previously reported methods.[9, 10] A summary of this method will be provided below. The first step towards being able to determine the triplet yields is determination of the triplet extinction coefficients.

i) Determination of triplet extinction coefficients of ACA derivatives

The triplet extinction coefficients were determined from triplet sensitisation experiments as described before. Briefly, PdOEP was used as triplet sensitiser and we found triplet energy transfer from PdOEP to ACA to occur within the first few microseconds, thereby outcompeting the triplet decay of neat PdOEP (>100 μs). As such the triplet transfer efficiency from PdOEP to ACA was treated as unity. As PdOEP is known to have near unity intersystem crossing yields, we estimate the formed population of triplets on the ACA derivatives to be equal to the initial population of singlet excited PdOEP molecules, i.e.:

$$[S_{1,\mathrm{PdOEP}}] \approx [\mathrm{T}_{1,\mathrm{ACA}}]$$

The steps to determine the triplet extinction coefficient are:

1. Measure the power absorbed by the sample P_{abs}: Subtract the power after the sample and reflected by the sample from the power before the sample.

2. Determine the number of absorbed photons per pulse by dividing P_{abs} by the energy per photon and the repetition rate of the laser:

$$\text{photons absorbed per pulse} = \frac{P_{\text{abs}}}{\left({hc}/{\lambda}\right) \text{ x 'laser rep rate'}}$$

3. Calculate the absorption volume V from the length of the cuvette l and the diameter of the pump pulse d as obtained from a beam profiler:

$$V = l\pi \left(\frac{d}{2}\right)^2$$

4. Determine the molar concentration of triplet states:

$$[\text{S}_{1,\text{PdOEP}}] = [\text{T}_{1,\text{ACA}}] = \frac{\text{photons absorbed per pulse}}{V \text{ x } N_{\text{A}}}$$

5. The differential transmission ${\Delta T}/{T}$ at the wavelength corresponding to the peak of the ACA triplet is taken from the plateau of the sensitisation data (averaged over a 10 nm interval) and converted to a change in absorbance ΔA:

$$\Delta A = -\log_{10}\left(\frac{\Delta T}{T} + 1\right)$$

6. ΔA is subsequently divided by the concentration of triplet states $[\text{T}_1]$ and the pathlength to get the triplet extinction coefficient ε_{T_1} in units of M^{-1} cm^{-1}:

$$\varepsilon_{\text{T}_1} = \frac{\Delta A}{l \text{ x } [\text{T}_1]}$$

ACA isomer	Triplet extinction coefficient (M^{-1} cm^{-1})
1-ACA	8 495
2-ACA	13 400
9-ACA	10 330

Table S4. Experimentally obtained triplet extinction coefficients of the studied ACA isomers under 532 nm excitation.

ii) Calculation of triplet yields

Having determined the triplet extinction coefficients, we subsequently calculated the triplet yields according to the following workflow:

1. The concentration of singlet excited states [S_1] was determined following steps 1-4 from the workflow to determine triplet extinction coefficients.
2. The peak triplet yield is determined from the peak value of $\Delta T/T$ of the ACA triplet photoinduced absorption (10 nm interval) from the nanosecond transient absorption spectroscopy data presented in Figure 3.
3. This differential transmission is converted to a ΔA value according to the formula presented in step 5 of the triplet extinction coefficient workflow.
4. The triplet concentration [T_1] is calculated:

$$[\mathrm{T_1}] = \frac{\Delta A}{l \text{ x } \varepsilon_{\mathrm{T_1}}}$$

5. The triplet yield Φ_{T} is calculated:

$$\Phi_{\mathrm{T}} = 100 \text{ x } \frac{[\mathrm{T_1}]}{[\mathrm{S_1}]}$$

Further Transient Absorption Spectroscopy

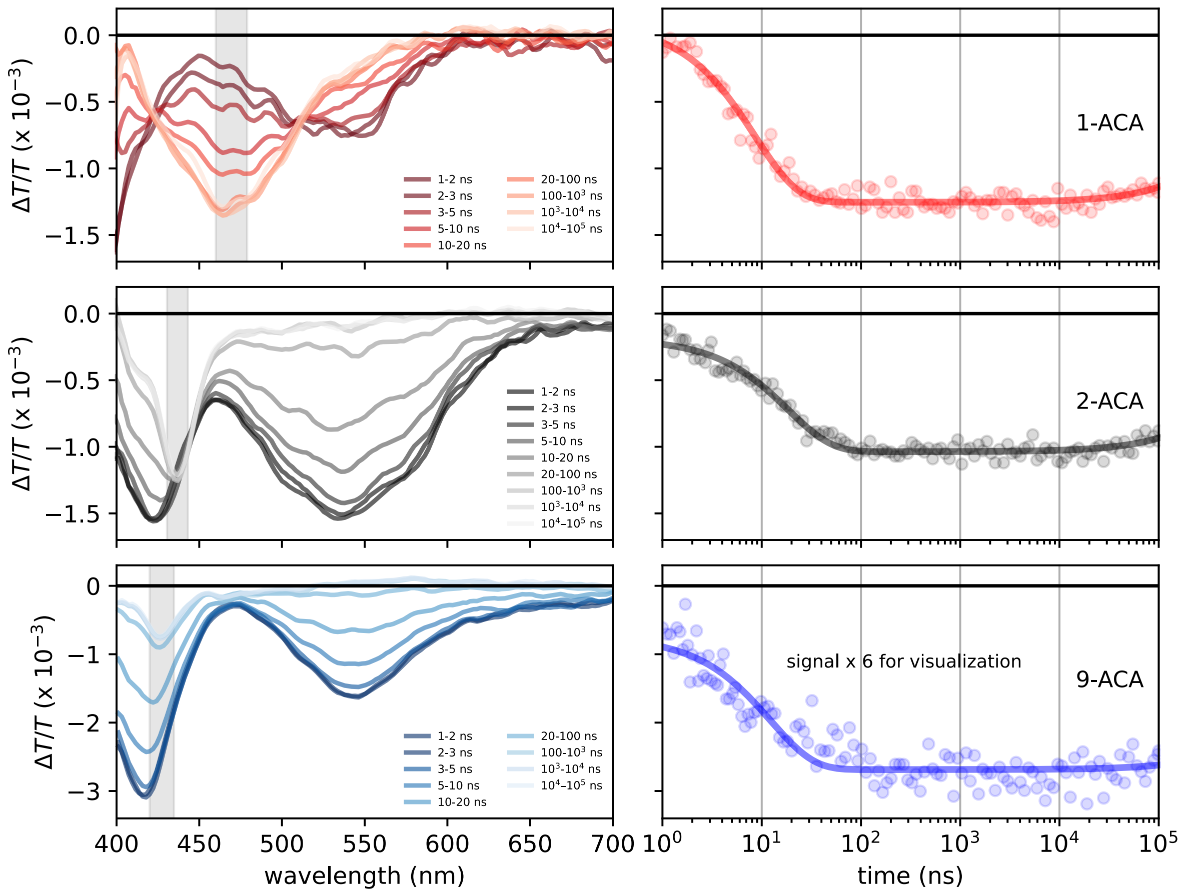

Figure S10. Long-time transient absorption spectra of the ACA isomers, λ_{ex} = 355 nm, and kinetics of the triplet photoinduced absorption.

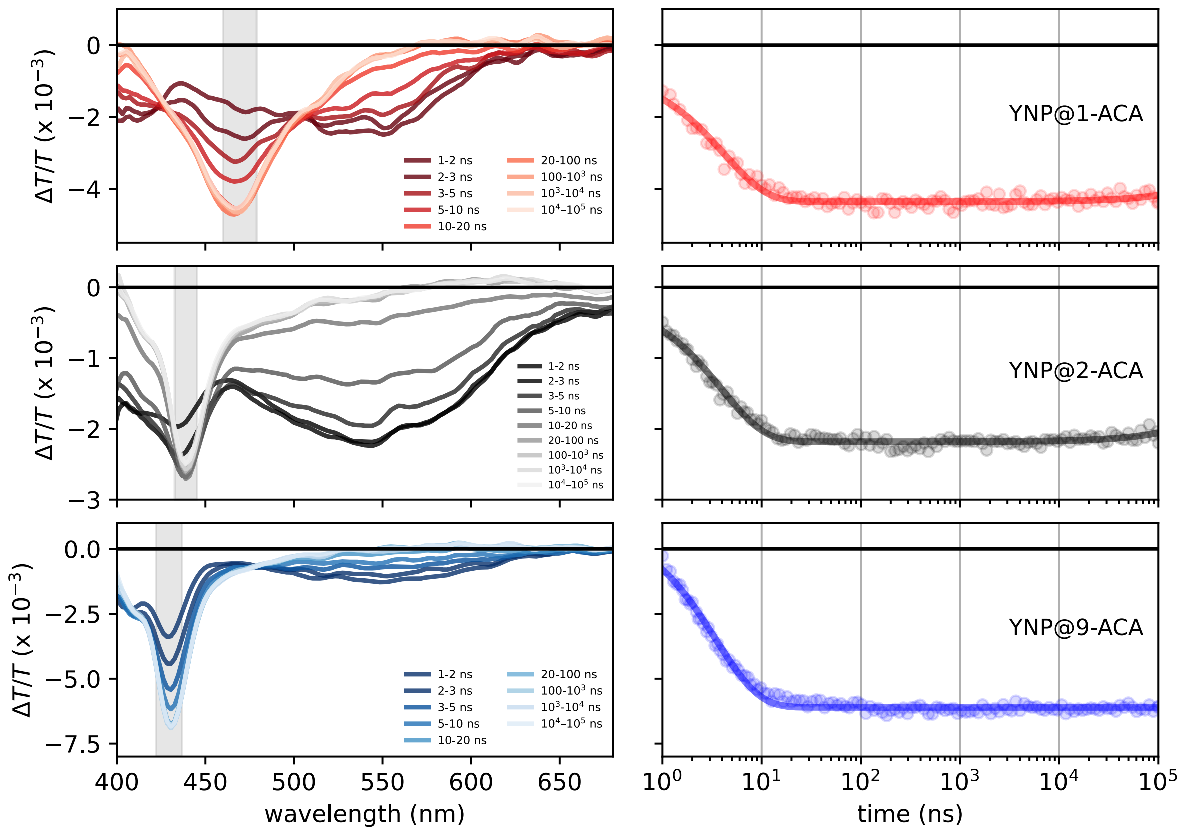

Figure S11. Long-time transient absorption spectra of the YNP@ACA nanohybrids, λ_{ex} = 355 nm, and kinetics of the triplet photoinduced absorption.

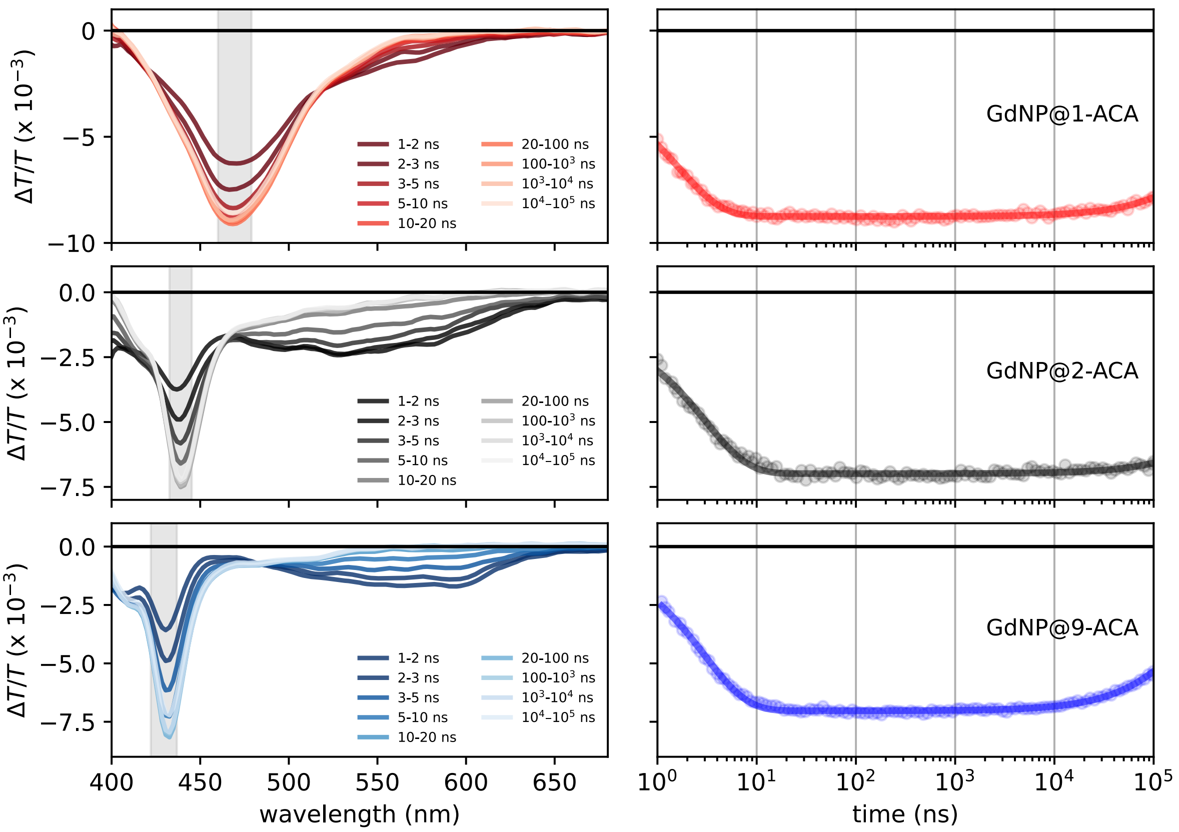

Figure S12. Long-time transient absorption spectra of the GdNP@ACA nanohybrids, λ_{ex} = 355 nm, and kinetics of the triplet photoinduced absorption.

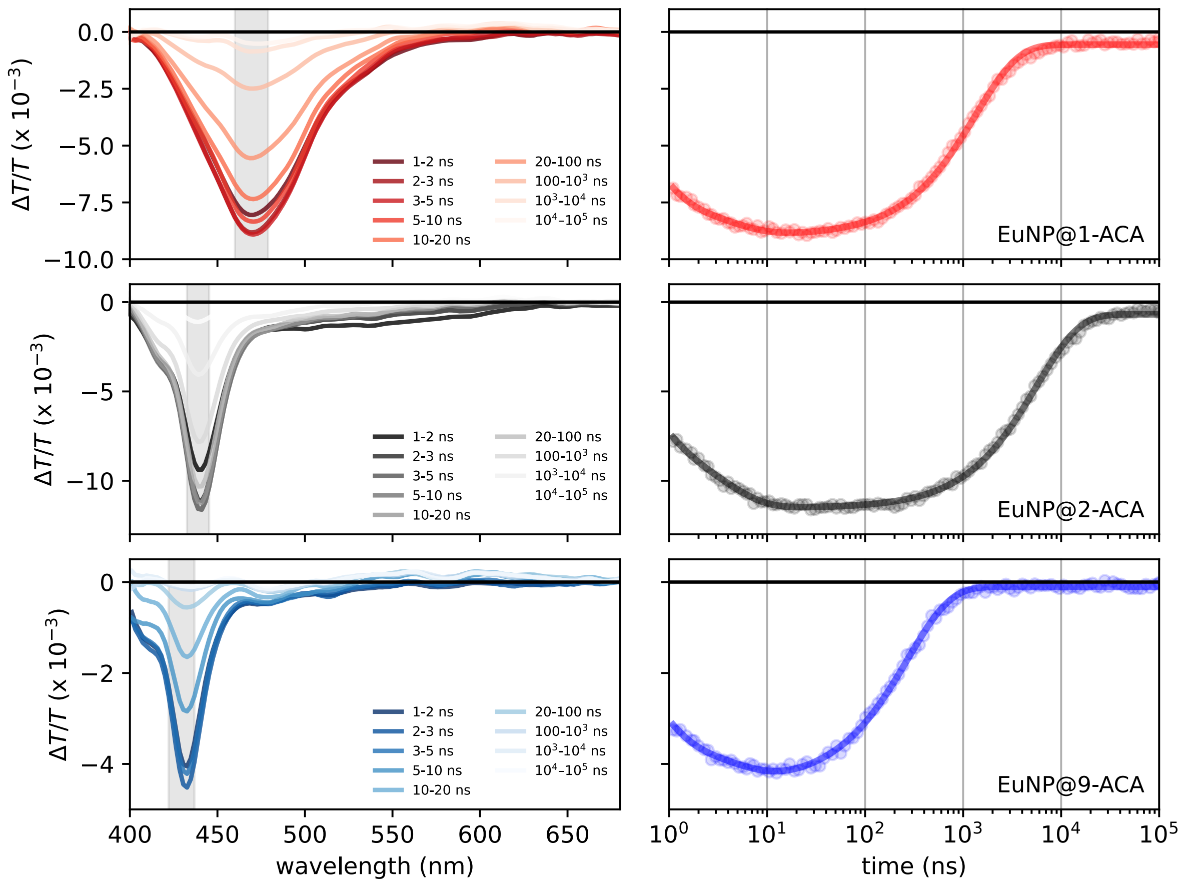

Figure S13. Long-time transient absorption spectra of the EuNP@ACA nanohybrids, λ_{ex} = 355 nm, and kinetics of the triplet photoinduced absorption.

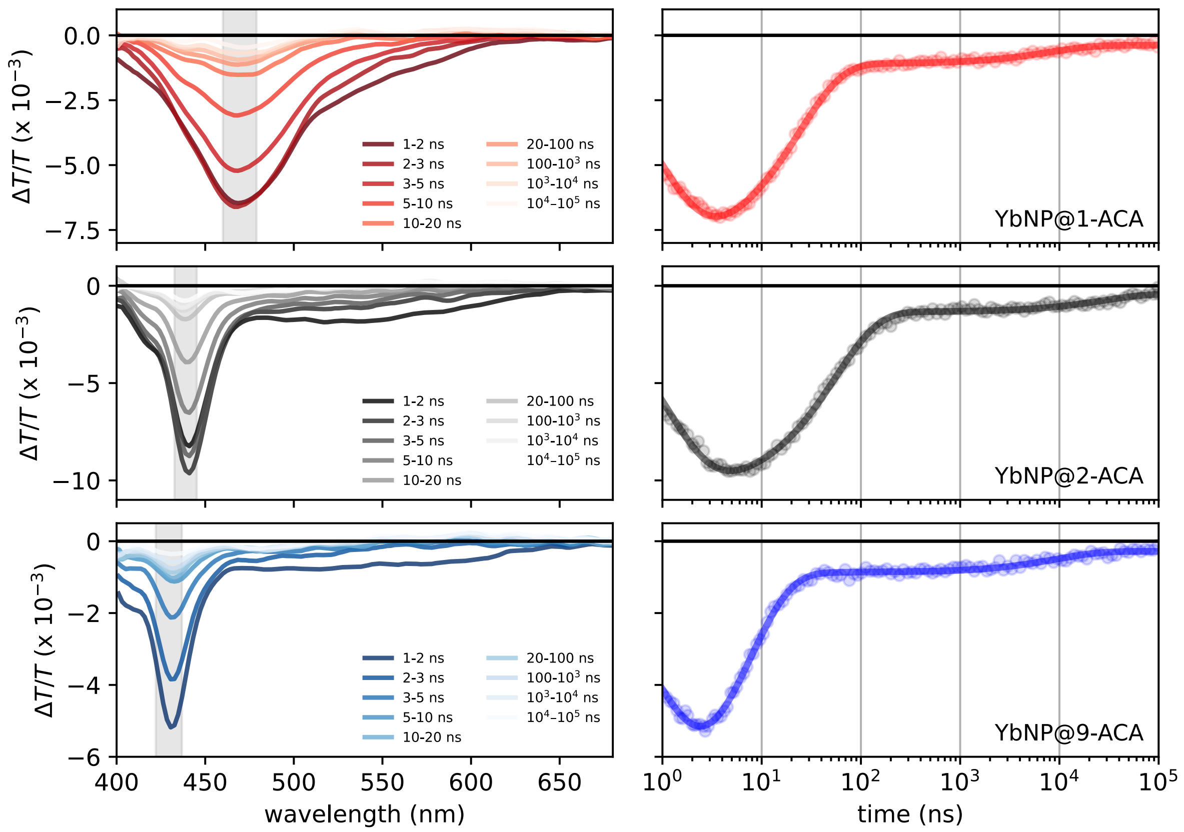

Figure S14. Long-time transient absorption spectra of the YbNP@ACA nanohybrids, λ_{ex} = 355 nm, and kinetics of the triplet photoinduced absorption.

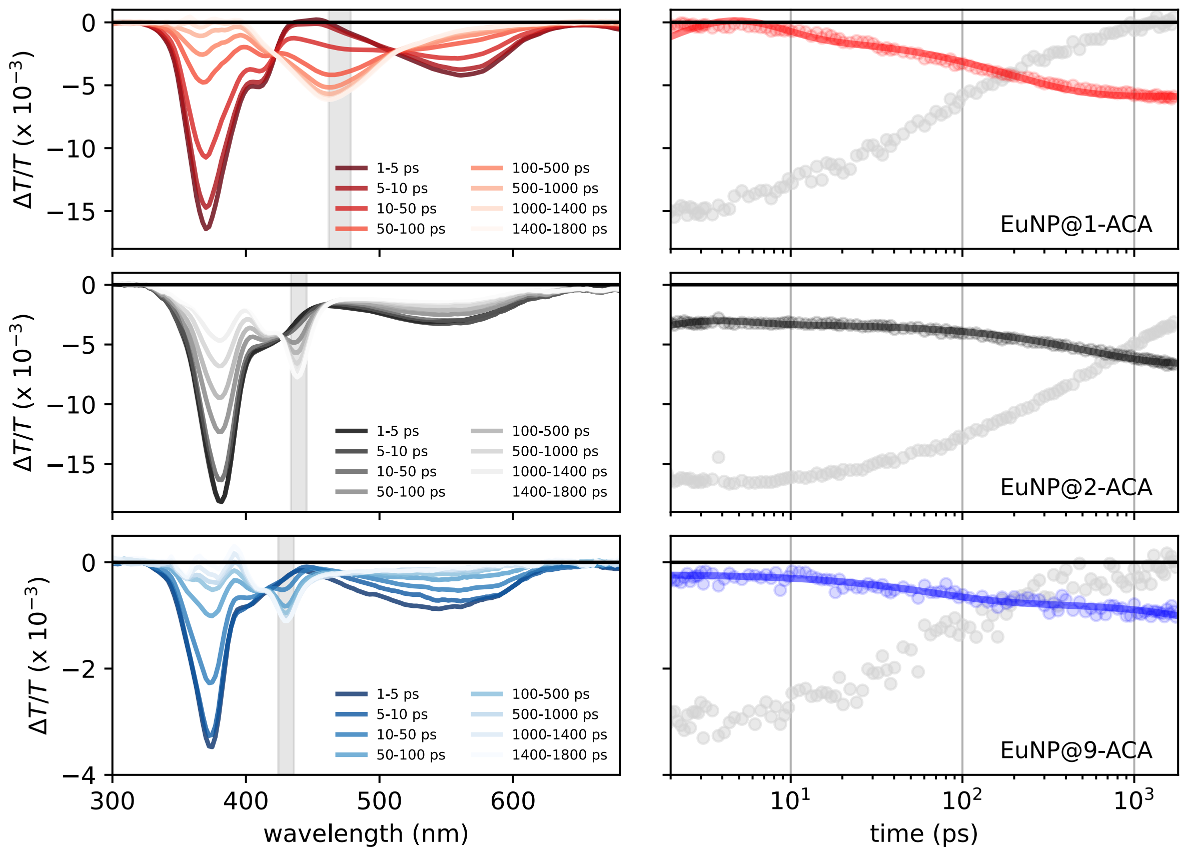

Figure S15. Short-time transient absorption spectra of the EuNP@ACA nanohybrids, λ_{ex} = 355 nm, and kinetics of the triplet photoinduced absorption (coloured lines) and singlet photoinduced absorption (grey lines).

Kinetic Modelling

The short time captured the first few ns which was sufficient to capture the singlet decay but not the triplet. Long time data up to 100 μs was stitched to the short time and was able to capture the triplet decay for most samples. A set of differential equations modelled the populations of singlet, triplet and lanthanide populations with shared (global) rate constants. The differential equations were integrated using the scipy package odeint and a wrapper function allowed lmfit's minimize package to perform a global fit of the rate constants via least squares minimisation. The lmfit package was used to enable facile modification of which parameters would be used for the fit and also produces parameter uncertainties based on the second derivative of fit quality.

Two different models were used to fit the various lanthanides which were dependent on the respective energy level structure with respect to the ACA S_1 and T_1 energy levels. YNP and GdNPs have no energy levels to accept energy from the ACA isomers, so these could be modelled with only 3 rate constants: k_{ISC}, k_{fl} + $k_{s,nr}$ (labelled $k_{s,other}$ in Figure S14 and the following ODEs), k_{ph} + $k_{t,nr}$ (labelled $k_{t,other}$ in Figure S14 and the following ODEs), see the bottom panel of Figure S14. The "other" rate constants refer to the combined radiative and non-radiative decay rates of the singlet and triplet, respectively (excluding the ISC rate), which are indistinguishable when analysing transient absorption data. From the YNP and GdNP fits, $k_{s,other}$ and $k_{t,other}$ were extracted and maintained constant for the subsequent analysis of EuNP@ACA and YbNP@ACA nanohybrids as we do not expect the majority of radiative and non-radiative deactivation rates to vary drastically from one lanthanide to another. In fact, we do expect the radiative rate of the triplet state through phosphorescence to vary depending on the specific lanthanide. However, as $k_{t,other}$ was 4 orders of magnitude smaller than k_{TET}, variation of this parameter by orders of magnitude made little difference to the other fitted parameters.

EuNP@ACA and YbNP@ACA nanohybrids can be modelled using a one or two level lanthanide system, respectively. Yb(III) only has one excited state energy level and as such a one state system is justified (middle panel of Figure S14). Eu(III) has many excited state energy levels and thus the model used is an oversimplification of the many lanthanide levels that exist and partake in interactions (top panel of Figure S14). However, as no lanthanide signals can be seen in (ordinary) transient absorption

spectra directly, we only include the minimum number of lanthanide energy levels necessary: the two level system is based on one lanthanide energy level interacting with the anthracene singlet and one with the triplet. Back transfer from the highest Eu(III) energy level to the ACA T_1 state was enabled.

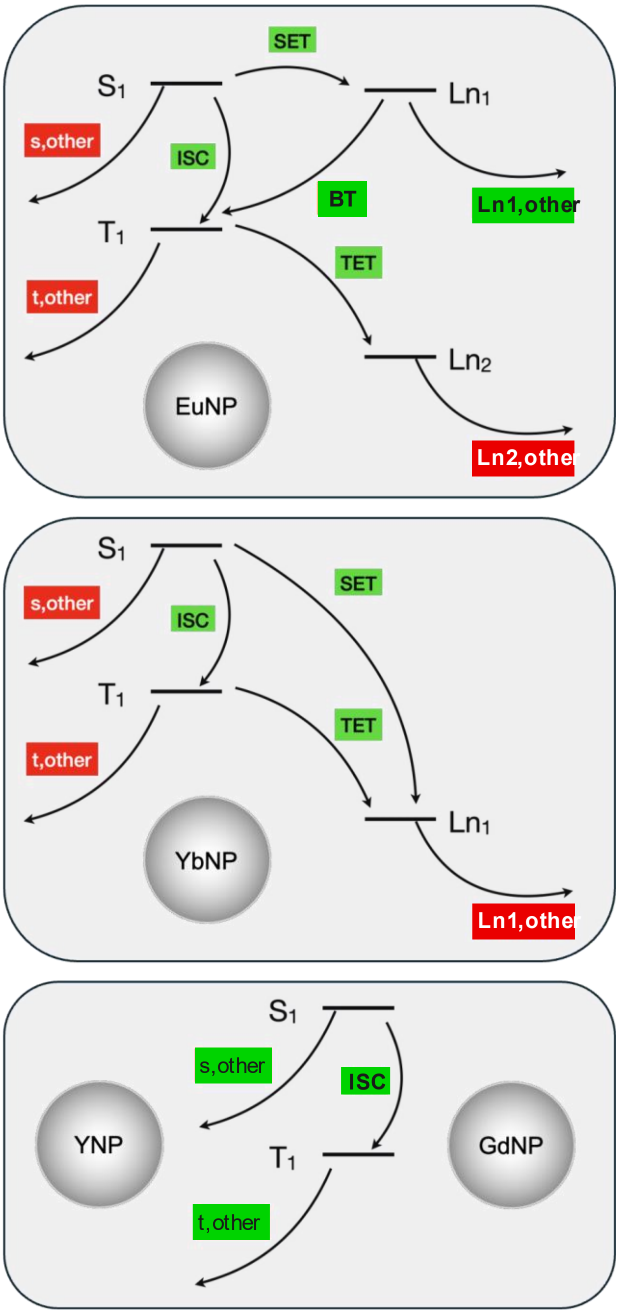

Figure S16. Overview of the kinetic models used to fit the ACA excited state dynamics in EuNP@ACA, YbNP@ACA, YNP@ACA and GdNP@ACA nanohybrids. Parameters shown in green boxes were allowed to vary in fits. Parameters in red boxes were kept constant for the fits.

Coupled differential equations used to fit the EuNP@ACA nanohybrid dynamics:

$$\frac{d[S_1]}{dt} = -\left(k_{\mathrm{SET}} + k_{\mathrm{ISC}} + k_{\mathrm{s,other}}\right) \times [S_1]$$

$$\frac{d[T_1]}{dt} = k_{\mathrm{ISC}} \times [S_1] + k_{\mathrm{BT}} \times [Ln_1] - \left(k_{\mathrm{TET}} + k_{\mathrm{t,other}}\right) \times [T_1]$$

$$\frac{d[Ln_1]}{dt} = k_{\mathrm{SET}} \times [S_1] - \left(k_{\mathrm{BT}} + k_{\mathrm{Ln1,other}}\right) \times [Ln_1]$$

$$\frac{d[Ln_2]}{dt} = k_{\mathrm{TET}} \times [T_1] - k_{\mathrm{Ln2,other}} \times [Ln_2]$$

Coupled differential equations used to fit the YbNP@ACA nanohybrid dynamics:

$$\frac{d[S_1]}{dt} = -\left(k_{\mathrm{SET}} + k_{\mathrm{ISC}} + k_{\mathrm{s,other}}\right) \times [S_1]$$

$$\frac{d[T_1]}{dt} = k_{\mathrm{ISC}} \times [S_1] - \left(k_{\mathrm{TET}} + k_{\mathrm{t,other}}\right) \times [T_1]$$

$$\frac{d[Ln_1]}{dt} = k_{\mathrm{SET}} \times [S_1] + k_{\mathrm{TET}} \times [T_1] - k_{\mathrm{Ln1,other}} \times [Ln_1]$$

Coupled differential equations used to fit the YNP@ACA and GdNP@ACA nanohybrid dynamics:

$$\frac{d[S_1]}{dt} = -\left(k_{\mathrm{ISC}} + k_{\mathrm{s,other}}\right) \times [S_1]$$

$$\frac{d[T_1]}{dt} = k_{\mathrm{ISC}} \times [S_1] - k_{\mathrm{t,other}} \times [T_1]$$

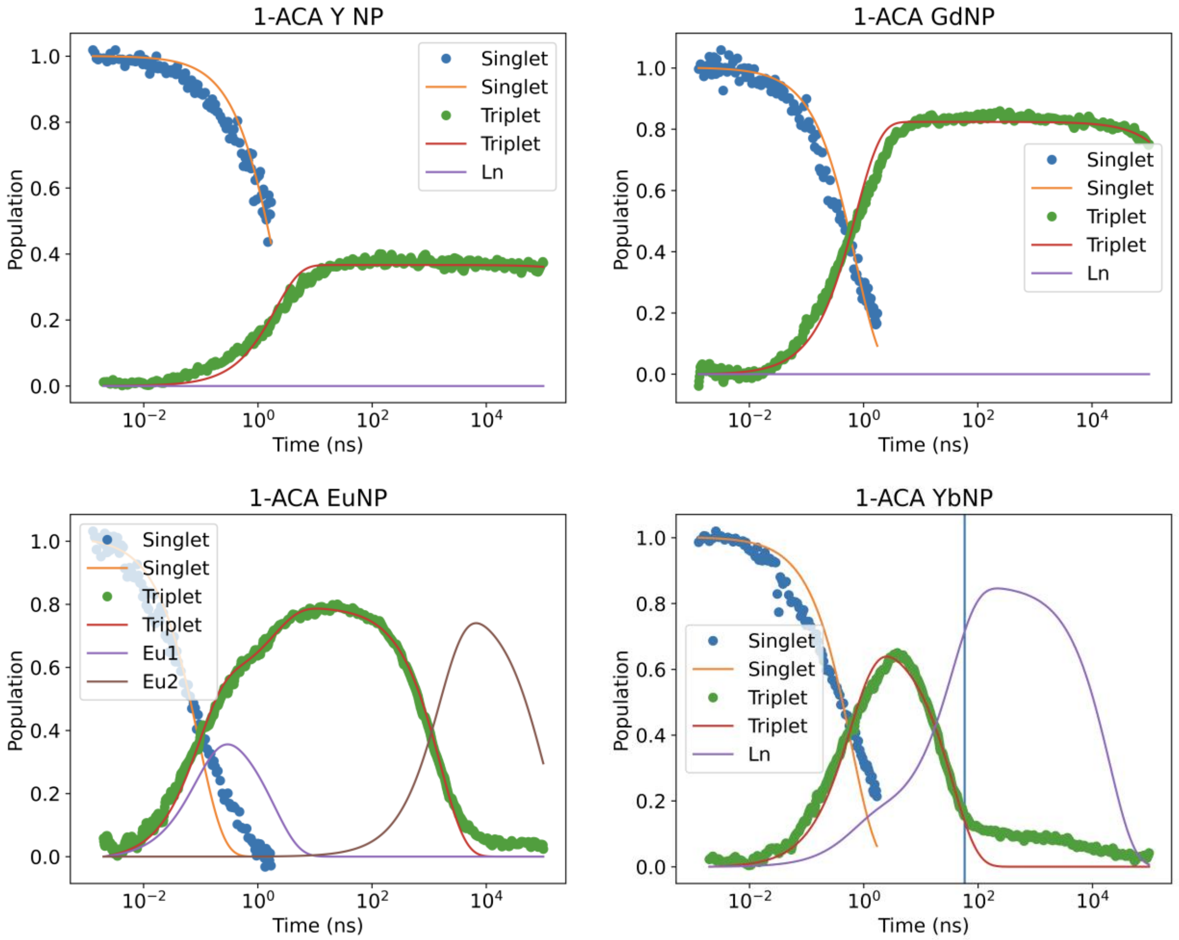

Figure S17. Kinetic modelling of the transient absorption spectroscopy kinetics of the coordinated 1-ACA isomer. Circular dots represent the experimentally obtained data, solid lines are the fits obtained through kinetic modelling. The vertical line in the 1-ACA YbNP plot indicates the time point up until which data for fitting was included.

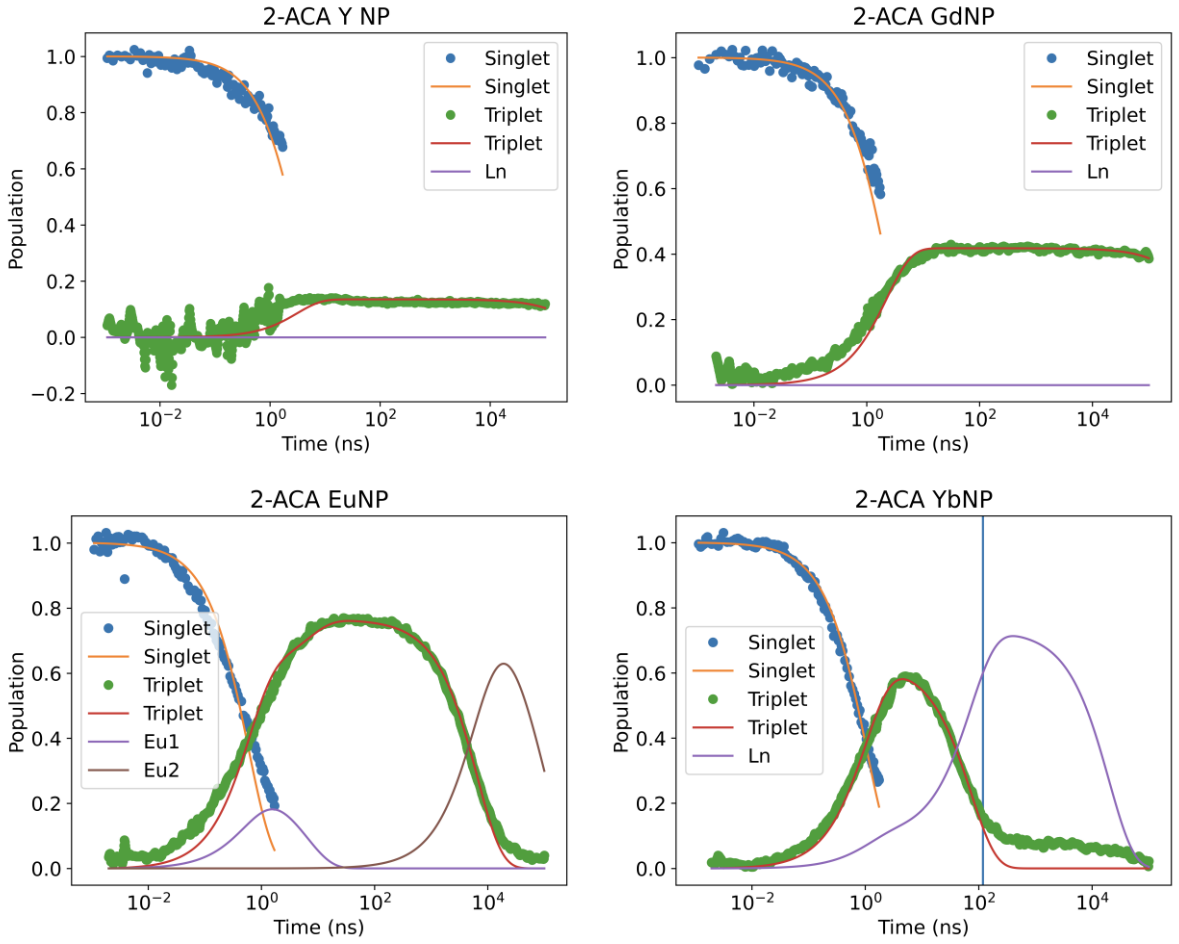

Figure S18. Kinetic modelling of the transient absorption spectroscopy kinetics of the coordinated 2-ACA isomer. Circular dots represent the experimentally obtained data, solid lines are the fits obtained through kinetic modelling. The vertical line in the 2-ACA YbNP plot indicates the time point up until which data for fitting was included.

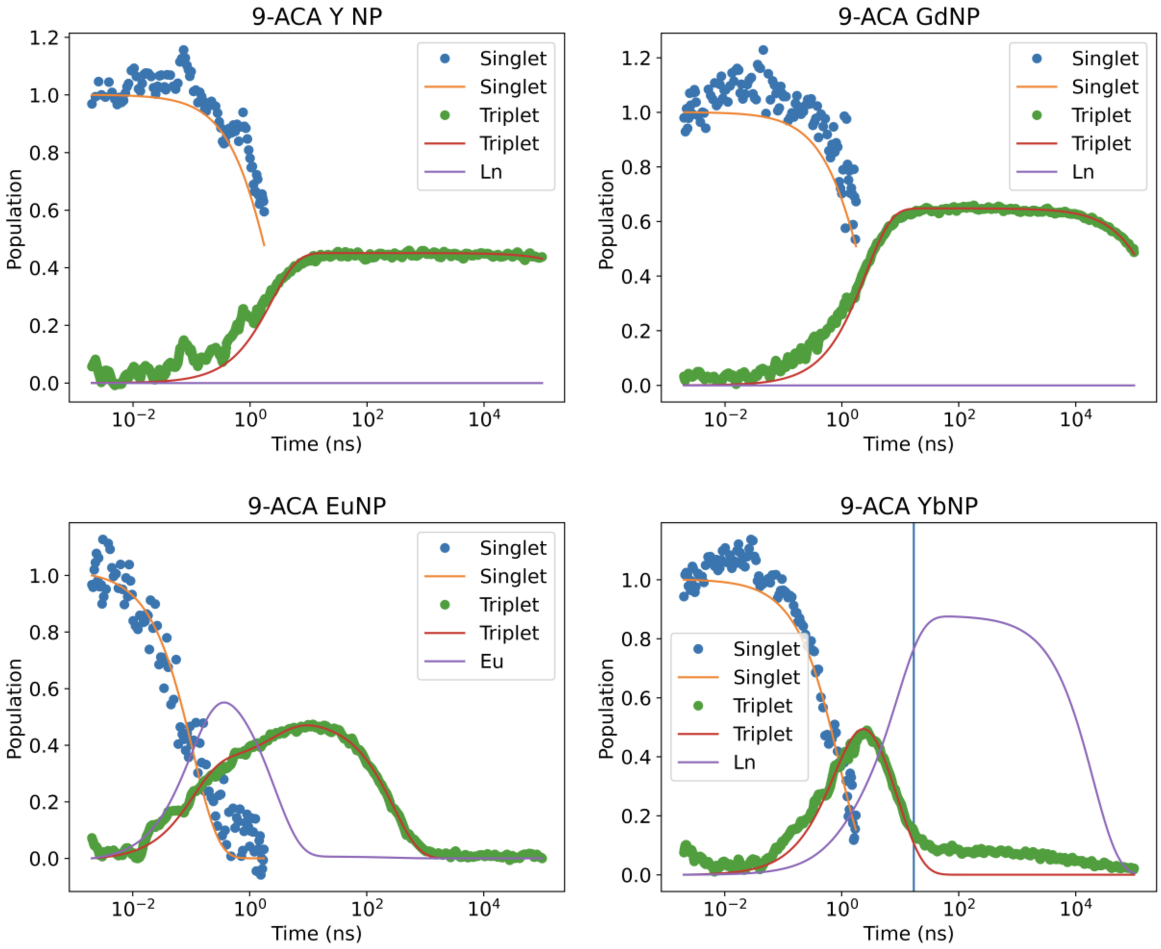

Figure S19. Kinetic modelling of the transient absorption spectroscopy kinetics of the coordinated 9-ACA isomer. Circular dots represent the experimentally obtained data, solid lines are the fits obtained through kinetic modelling. The vertical line in the 9-ACA YbNP plot indicates the time point up until which data for fitting was included.

Orientation of ACA Isomers

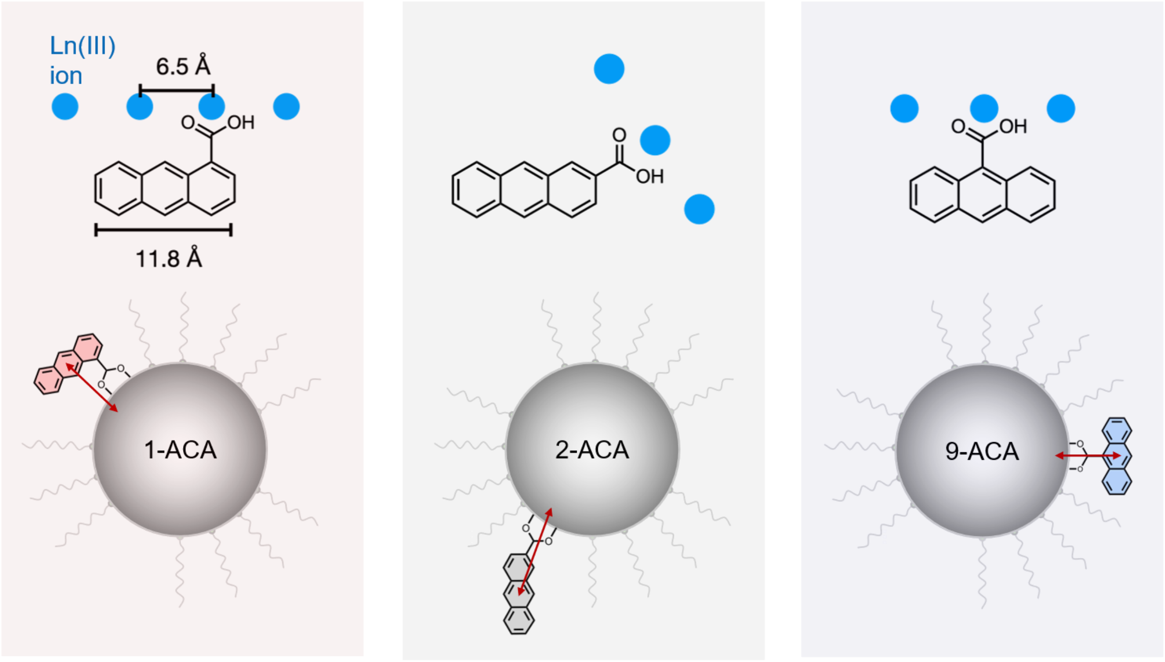

Figure S20. Expected orientations of the ACA isomers on the surface of the nanoparticles and their interactions with lanthanide ions on the nanoparticle surface.

References

(1) Wang, F.; Deng, R.; Liu, X. Preparation of core-shell $NaGdF_4$ nanoparticles doped with luminescent lanthanide ions to be used as upconversion-based probes. *Nat. Protoc.* **2014**, *9* (7), 1634-1644.

(2) de Mello, J. C.; Wittmann, H. F.; Friend, R. H. An improved experimental determination of external photoluminescence quantum efficiency. *Adv. Mater.* **1997**, *9* (3), 230-232.

(3) Gélinas, S.; Paré-Labrosse, O.; Brosseau, C.-N.; Albert-Seifried, S.; McNeill, C. R.; Kirov, K. R.; Howard, I. A.; Leonelli, R.; Friend, R. H.; Silva, C. The binding energy of charge-transfer excitons localized at polymeric semiconductor heterojunctions. *J. Phys. Chem. C* **2011**, *115* (14), 7114-7119.

(4) Johnson, N. J.; Oakden, W.; Stanisz, G. J.; Scott Prosser, R.; van Veggel, F. C. Size-tunable, ultrasmall $NaGdF_4$ nanoparticles: insights into their T_1 MRI contrast enhancement. *Chem. Mat.* **2011**, *23* (16), 3714-3722.

(5) Schweitzer, C.; Schmidt, R. Physical mechanisms of generation and deactivation of singlet oxygen. *Chem. Rev.* **2003**, *103* (5), 1685-1758.

(6) Wang, X.; Jiang, C.; Wang, Z.; Cohen, B. E.; Chan, E. M.; Chen, G. Triplet-induced singlet oxygen photobleaches near-infrared dye-sensitized upconversion nanosystems. *Nano Lett.* **2023**, *23* (15), 7001-7007.

(7) Lu, T.; Chen, F. Multiwfn: A multifunctional wavefunction analyzer. *J. Comput. Chem.* **2012**, *33* (5), 580-592.

(8) Gao, X.; Bai, S.; Fazzi, D.; Niehaus, T.; Barbatti, M.; Thiel, W. Evaluation of spin-orbit couplings with linear-response time-dependent density functional methods. *J. Chem. Theory Comput.* **2017**, *13* (2), 515-524.

(9) van Turnhout, L.; Congrave, D. G.; Yu, Z.; Arul, R.; Dowland, S. A.; Sebastian, E.; Jiang, Z.; Bronstein, H.; Rao, A. Distance-independent efficiency of triplet energy transfer from π-conjugated organic ligands to lanthanide-doped nanoparticles. *J. Am. Chem. Soc.* **2024**, *146* (32), 22612-22621.

(10) Millington, O.; Montanaro, S.; Leventis, A.; Sharma, A.; Dowland, S. A.; Sawhney, N.; Fallon, K. J.; Zeng, W.; Congrave, D. G.; Musser, A. J. Soluble diphenylhexatriene dimers for intramolecular singlet fission with high triplet energy. *J. Am. Chem. Soc.* **2023**, *145* (4), 2499-2510.
